# Supplementary material for: Underwater CAM photosynthesis elucidated by Isoetes genome
Source: Nat Commun. 2021 Nov 3;12:6348. doi: 10.1038/s41467-021-26644-7 (PMC8566536; doi:10.1038/s41467-021-26644-7)
Supplement: Supplementary file 1 — Supplementary Information [file 41467_2021_26644_MOESM1_ESM.pdf]

Supplementary information for

Underwater CAM photosynthesis elucidated by *Isoetes* genome

Wickell *et al.*

Supplementary Note 1. RNA editing in the *Isoetes taiwanensis* chloroplast genome

The mitochondrial genome of *Isoetes engelmannii* was previously shown to have one of the highest numbers of RNA-editing sites known to date[1], in which a total of 1,782 sites were affected, and both the canonical C-to-U and the reverse U-to-C edits were found. However, it is unclear if the plastid genome of *Isoetes* also exhibits a similar level of RNA-editing. Here we mapped our RNA-seq data (that were not poly-A enriched) to the *I. taiwanensis* plastome, and identified a total of 465 RNA-editing sites. While 43 editing sites were located in noncoding regions, 422 were found in 70 protein coding genes. Of these, the majority were of the C-to-U type (399 sites) with U-to-C editing found at 23 sites. The total number of RNA-editing sites in *Isoetes* is comparable to plastomes of other seed-free plants, but substantially lower than *Selaginella*, where more than 3,400 sites were reported[2]. There appears to be a bias toward edits at the first codon position with 233, 56, and 122 editing events found at the first, second, and third positions respectively. Despite this, slightly more than half of edits (54%), including all identified U to C edits, were silent. The most heavily edited gene was *petN* with editing detected in 5.6% of its nucleotides. In addition, *psb* genes were generally highly modified, comprising 5 of the 10 most edited genes (Supplementary Fig. 2). The reason behind such concentrated editing is unclear.

Supplementary Note 2. Gene family evolution

To assess gene homology between *I. taiwanensis* and other plants, we conducted an Orthofinder analysis containing 25 species from across the plant phylogeny. Our analysis placed 647,535 genes into 40,144 orthogroups. Of those, 12,890 orthogroups contained at least one gene from *I. taiwanensis* with 3,391 being unique. A total of 6,241 *I. taiwanensis* genes are singletons that were not assigned to any group. Surprisingly, *I. taiwanensis* genes showed greater orthogroup overlap with seed plants than with any seed-free taxa (Supplementary Fig. 4). While this relationship may have been driven by the relatively high number of angiosperm taxa included in the original analysis relative to other groups, a similar trend was not seen in *Selaginella moellendorffii* (Supplementary Fig. 4).

Supplementary Note 3. Genes related to lignin biosynthesis

One of the foremost features of vascular plants is lignification. The three major types of lignin monomers (hydroxyphenyl, guaiacyl, and syringyl; H-, G-, and S-lignin) are sourced from the phenylpropanoid pathway, and lycophytes use intriguing biochemical routes towards these compounds. In *Selaginella moellendorffii*, S-lignin is produced via two enzymes, CAFFEIC ACID/5-HYDROXYFERULIC ACID O-METHYLTRANSFERASE (SmCOMT) and FERULATE 5-HYDROXYLASE (SmF5H); both evolved independently from the canonical angiosperm COMT and F5H counterparts[3,4]. Similar to *S. moellendorffii*, *I. taiwanensis* lacks canonical *COMT* and *F5H* genes, but has orthologs of *SmCOMT* and *SmF5H* as well as the rest of lignin biosynthesis enzymes—offering putative routes towards all types of lignin (Supplementary Figs. 5-17). This result implies that the evolution of such an alternative S-lignin pathway might predate the divergence of *Selaginella* and *Isoetes*. However, the presence of S-lignin in *Isoetes* species

appears ambiguous[5]. Residue analyses of *I. taiwanensis COMT* candidates found that, in contrast to the functionally characterized *SmCOMT* (Smoel_227279), the catalytic triad (HDE) contains a radical substitution (HNE) (Supplementary Fig. 12); further, in contrast to all other gene families (Supplementary Figs. 12 and 17), none of the *ItCOMT* homologs showed detectable gene expression. Future work on lignin biochemistry and enzyme assays are needed to clarify the evolution of S-lignin biosynthesis in lycophytes.

Supplementary Note 4. Genes related to stomata development

The presence of genes associated with stomatal development in *Isoetes* is of interest because the production of functional stomata varies throughout the genus. Some aquatic and at least one terrestrial species do not produce stomata or have non-functional stomata occluded by wax[6]. Amphibious species, such as *I. taiwanensis*, do produce functional stomata under aerial conditions[6], but not when they are submerged. It is important to note that stomatal control is thus not relevant in aquatic CAM plants.

A recent study using transcriptomic data found evidence for loss of stomatal patterning genes *EPIDERMAL PATTERNING FACTOR 1/2* (*EPF1/2*) and *TOO MANY MOUTHS* (*TMM*)[7], even though this species is known to produce stomata[8]. In *I. taiwanensis*, while our genomic evidence corroborated the absence of *EPF1/2* orthologues, we did find two copies of *TMM*, one of which was expressed at low levels in leaf tissue (Supplementary Fig. 18). In addition, we confirmed the presence of orthologues of other stomatal patterning genes, such as *SPEECHLESS/MUTE/FAMA* (*SMF*) and *FAMA* (Supplementary Fig. 19). Thus, despite their aquatic growth habit, *Isoetes* appear to have retained much of the genetic machinery required for stomatal development (Supplementary Table 1). This is what we might expect given *Isoetes*' amphibious nature, and highlights the danger of inferring gene absence from transcriptomic data alone.

Supplementary Note 5. Genes related to root development

The homology of *Isoetes* roots has been the source of some controversy. *Isoetes* 'rootlets' are highly similar to the 'stigmarian' roots of ancient lycopsids and their superficial resemblance to aboveground structures has led some researchers to speculate that *Isoetes* 'rootlets' are in fact modified leaves[9]. However, a recent study[10] of the *I. echinospora* transcriptome identified *ROOT HAIR DEFECTIVE SIX-LIKE* (*RSL*) genes, which have been used as a marker for root development in vascular plants[11,12]. We found multiple copies of *RSL* genes in the *I. taiwanensis* genome and subsequent phylogenetic analysis placed 5 copies in the RSL Class I clade and a single copy in the RSL Class II clade (Supplementary Fig. 20). Taken together, our results are consistent with the earlier transcriptome-based study and provide evidence for homology of root structures across vascular plants, at least at the genetic level.

Supplementary Note 6. Isoetes time-of-day global gene expression

Time-of-day (TOD) gene expression underlies the fundamental separation of carbon capture during CAM photosynthesis and looking at the global regulation of the transcriptome

provides clues to the architecture of CAM in different plants[13–15]. We carried out a TOD time course In *I. taiwanensis* plants that were grown under light/dark cycles and constant temperature (LDHH) and were sampled every 3 hours for 27 hours. As expected in TOD time courses, the individual timepoints were separated in two-dimensional space consistent with the time they were sampled, which results in a clockwise representation of expression (Supplementary Fig. 25). Replicates also cluster close together consistent with a high-quality time course.

Cycling genes were predicted using a model fitting approach implemented in HAYSTACK[16], which resulted in the identification of 3,241 cycling genes that represented 10% of the expressed genes (Supplementary Fig. 27a,b). Genes displayed peak expression, or phase, at every hour over the day, with two pronounced concentrations of genes at dawn (Zeitgeber 0; ZT0) and at dusk (ZT14) (Supplementary Fig. 27a). In general in flowering plants, a concentration of genes with peak expression at or before dawn (lights-on) has been observed[13,17–27]. The concentration of cycling genes near dawn and dusk is consistent with the circadian clock and TOD expression playing a role in synchronizing biology so that the organism can anticipate daily environmental changes, which enhances fitness[28,29].

TOD regulated expression ensures biological processes are phased to specific times over the day[17]. We identified significant gene ontology (GO) terms at every hour over the day and then plotted these terms to highlight that they are TOD-specific (Supplementary Fig. 27c; Supplementary Data 4). In general, *I. taiwanensis* is similar to other organisms with chloroplast machinery ramping up before dawn, photosynthesis pathways active at dawn, energy sensing pathway midday, response to the biotic and abiotic environment at dusk, and cellular processes occur in the evening. We did note various transport related GO terms in genes with peak expression midday (ZT=05) suggesting they may be ramping up to anticipate the dark phase of the day for CAM photosynthesis. Enrichment of transport related GO terms was primarily driven by TOD expression of three vacuolar ATPase genes: Itaiw_v1_scaffold_100_g37807, Itaiw_v1_scaffold_19_g14206, and Itaiw_v1_scaffold_7_g06931. Vacuolar ATPase is essential to establishing an H^+ gradient that in turn, drives transport of malate across the vacuolar membrane[13]. Unfortunately, no other transport proteins that could be clearly linked to malate transport were found in this cluster. Likewise, ALMTs which have been indicated as important drivers of vacuolar malate influx and efflux in pineapple (*Ananas*)[14] and *Kalanchoe*[15] did not exhibit TOD expression profiles in *I. taiwanensis*.

Supplementary Note 7. Comparison of *Isoetes'* TOD gene expression with other land plants

The number of genes controlled in a TOD fashion in *I. taiwanensis* is on the low side (10%) compared to most other plants tested to date[17,21,30]. Therefore, we wanted to see if there were commonalities of the *I. taiwanensis* cycling genes to species with high quality data or that were closely related. *Arabidopsis* has the most diurnal and circadian time courses available of any species ranging from different conditions to different tissues and genetic backgrounds. One study found that 90% of all *Arabidopsis* genes cycled under at least one of 11 environmental conditions suggesting that the type of external signal is important for the number of cycling genes detected[17]. Since we only looked at one condition of light/dark cycles and constant temperature (LDHH), we asked how the cycling orthologs compared between *I. taiwanensis* and *Arabidopsis* by condition. Since *I. taiwanensis* and *Arabidopsis* are distant evolutionarily, we

utilized reciprocal best BLAST hits (RBHs) between the two proteomes to approximate orthologs. There are 8,064 RBHs between *I. taiwanensis* and *Arabidopsis*, 1,280 (16%) of which cycle in *Isoetes* (Supplementary Fig. 28a). Overall, a higher percentage of both *Arabidopsis* and *I. taiwanensis* RBHs cycle compared to the global set of each, consistent with more highly conserved genes cycling in plants[25].

It has been shown that the closely related moss *Physcomitrium patens* and *Selaginella moellendorffii* have 18 and 39% of their genes under TOD regulation respectively[21]. Leveraging these datasets, we asked what the relationship between TOD peak expression between *I. taiwanensis* and these species. We found 8,565 and 8,724 RBHs between *P. patens* and between *S. moellendorffii*, and 851 and 1,050 shared cycling genes respectively. The differences in the TOD peak gene expression were spread out between *I. taiwanensis* and *S. moellendorffii* consistent with generally differently phased processes (Supplementary Fig. 28b); a similar trend was found between the 427 shared cycling genes between *I. taiwanensis* and *Arabidopsis* (LDHH condition). However, in contrast, *I. taiwanensis* and *P. patens* had a distinct phase difference with genes either sharing a similar phase or having antiphasic expression. One interpretation of this pattern is that *I. taiwanensis* and *P. patens* are similar in general in how they govern TOD information (in contrast to *S. moellendorffii*), yet since *I. taiwanensis* is a CAM plant some activities are phased to a distinct TOD. Antiphasic expression between *I. taiwanensis* and *P. patens* could also be the result of the potential *GI/PRR1* morning specific loop identified in *I. taiwanensis* (see below; Fig. 5). Additional experiments are required to better understand the TOD relationship across CAM and non-CAM plants.

Supplementary Note 8. TOD expression of core circadian clock genes

Central to global TOD expression is the cycling of the core circadian clock, which has been best worked out in the model plant *Arabidopsis*[31]. The plant circadian clock has both negative and positive feedback loops based on two gene families, the single MYB (sMYB) transcription factors and the *PSEUDO RESPONSE REGULATOR* (*PRR*). The sMYB family is made up of founding members *LATE ELONGATED HYPOCOTYL* (*LHY*)[32], *CIRCADIAN CLOCK ASSOCIATED 1* (*CCA1*)[33] and later the *REVEILLE* (*RVE*) family[34]. The *RVE* family is composed of 8 members: *RVE1*, *RVE2*, *RVE3*, *RVE4*, *RVE5*, *RVE6*, *RVE7* and *RVE8*; all of the sMYBs are expressed in the morning or midday[17]. The PRR family has 5 members: *PRR1*. *PRR3*, *PRR5*, *PRR7*, and *PRR9*. *PRR1* is the founding member that was identified in an early genetic screen and named *TIMING OF CAB1 EXPRESSION* (*TOC1*)[35]. The PRRs have peak expression over the entire day starting at dawn (*PRR9*), midday (*PRR7*), evening (*PRR3* and *PRR5*) and early night (*PRR1*)[17,36]. Another fundamental aspect of the circadian feedback loop are genes expressed at dusk or early in the dark phase such as *GIGANTEA* (*GI*) and the evening complex (EC); the EC is comprised of genes that are such as *EARLY FLOWERING 3* (*ELF3*), *ELF4* and *LUX ARRHYTHMO* (*LUX*)[31].

Since it has been shown that the phase of peak expression of core circadian clock genes is conserved from algae to higher plants[21,37,38], we looked at the cycling patterns of the *I. taiwanensis* circadian clock orthologs as an additional quality control of the time course and to identify potential differences in the core circadian clock. Potential orthologs were identified by searching our Orthofinder families using published core circadian clock genes across species. We found that the core circadian clock orthologs in *I. taiwanensis* displayed similar expression

such as the sMYB genes were expressed predominantly at dawn (*RVE*), while PRRs, *ELF3* and *LUX* were expressed midday to dusk (Supplementary Fig. 29). In addition, the expression of core clock genes was similar to the related lycophyte *S. moellendorffii*.

However, we did note some differences in the core clock components as well, such as *TOC1* and *GI* that in addition to having several extra copies compared to most genomes, also display an altered peak phase of expression (Fig. 5). It is possible that this may reflect a general difference in CAM plants. Therefore, we looked at the three other CAM plants *Sedum album*, *Kalanchoe fedtschenkoi* and *Ananas comosus* (pineapple) for which there is TOD transcriptome information. Most of the core circadian clock components cycled with a similar phase as other land plants and also similar to *I. taiwanensis*. Some phase differences between the obligate CAM plant *K. fedtschenkoi* and *Arabdiopsis* have been noted for some of the core clock genes[39], and some genes change phase between C3 and CAM in the facultative *S. album*[13]. However, neither obligate or facultative plants displayed the antiphasic expression for *GI* and *TOC1/PRR1* we observe in *I. taiwanensis* (Supplementary Fig. 30).

Supplementary Table 1. *Isoetes taiwanensis* has retained many genes essential to stomatal development.

Gene symbol	Copy number	*I. taiwanensis* gene IDs
SCRM/2	5	Itaiw_v1_scaffold_13_g10966, Itaiw_v1_scaffold_4_g04754, Itaiw_v1_scaffold_77_g34972, Itaiw_v1_scaffold_80_g35483, Itaiw_v1_scaffold_81_g35616
TMM	2	Itaiw_v1_scaffold_84_t35898, Itaiw_v1_scaffold_61_t31916
EPF1	0	NA
EPF2	0	NA
EPFL6	1	Itaiw_v1_contig_13_g42546, Itaiw_v1_contig_28_g43228, Itaiw_v1_contig_48_g43721, Itaiw_v1_contig_881_g47963, Itaiw_v1_scaffold_22_g15913, Itaiw_v1_scaffold_22_g15923, Itaiw_v1_scaffold_43_g26242, Itaiw_v1_scaffold_6_g06021
EPFL9	0	NA
SMF	3	Itaiw_v1_contig_32_t43316, Itaiw_v1_scaffold_5_t05017, Itaiw_v1_scaffold_57_t30612
FAMA	1	Itaiw_v1_scaffold_13_g11103
MUTE	0	NA

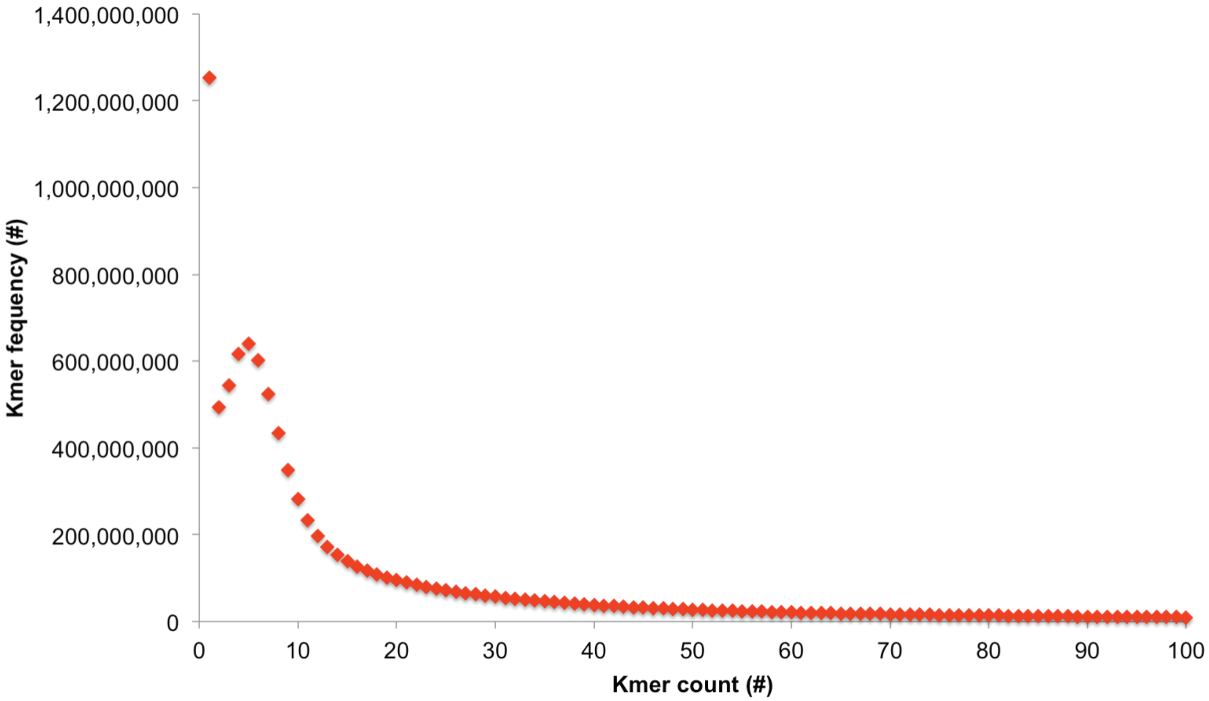

Supplementary Figure 1. *Isoetes taiwanensis* genome size estimate based on short read k-mer frequency. The genome size was estimated to be 1,647,045,703 bp by k-mer frequency (*k*=19) analysis using Illumina 2x150 bp short reads.

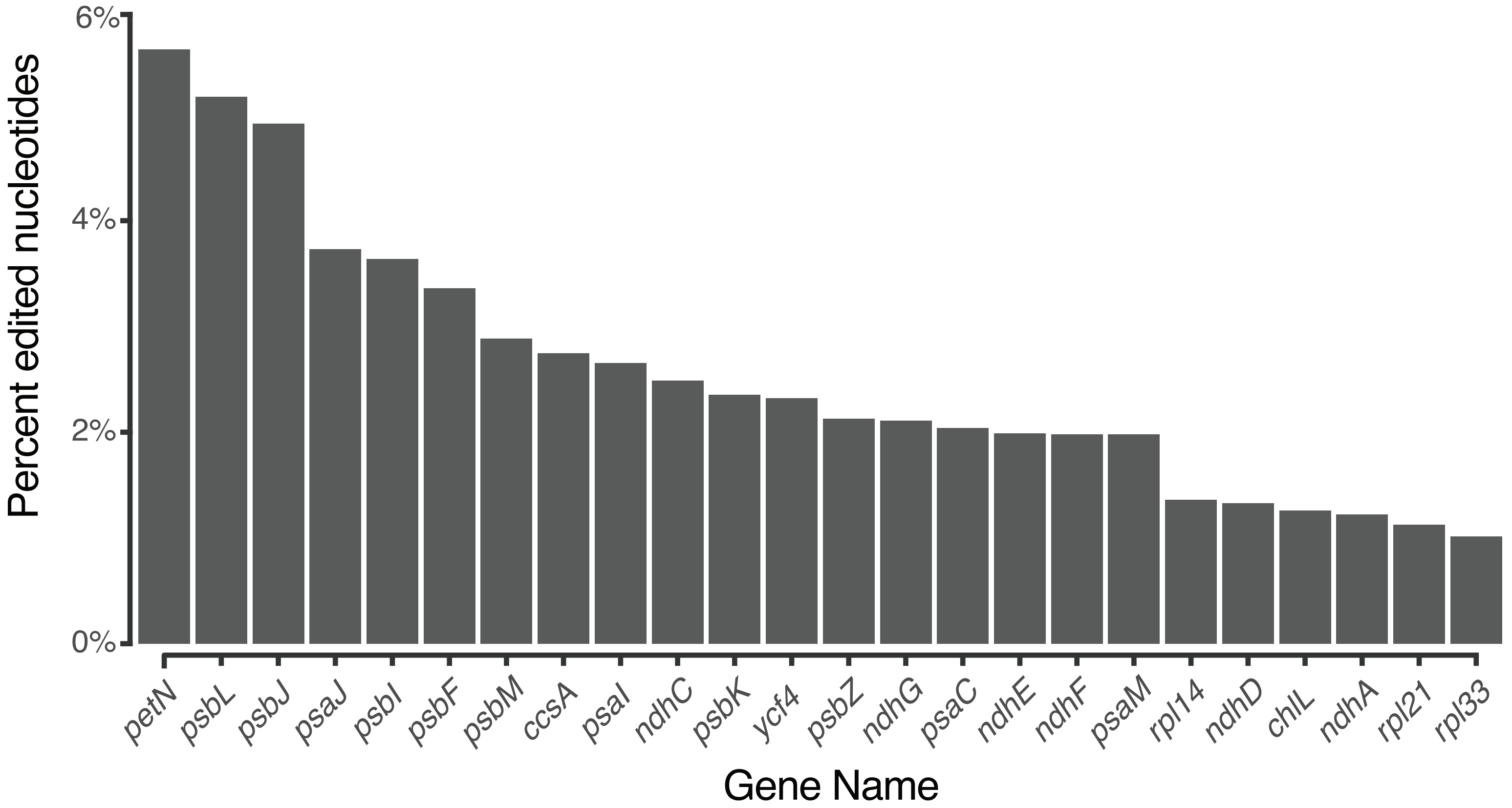

Supplementary Figure 2. The *Isoetes taiwanensis* plastome shows a high degree of RNA editing. A plot showing the number of editing sites as a percentage of gene length in genes with greater than 1% edited nucleotides.

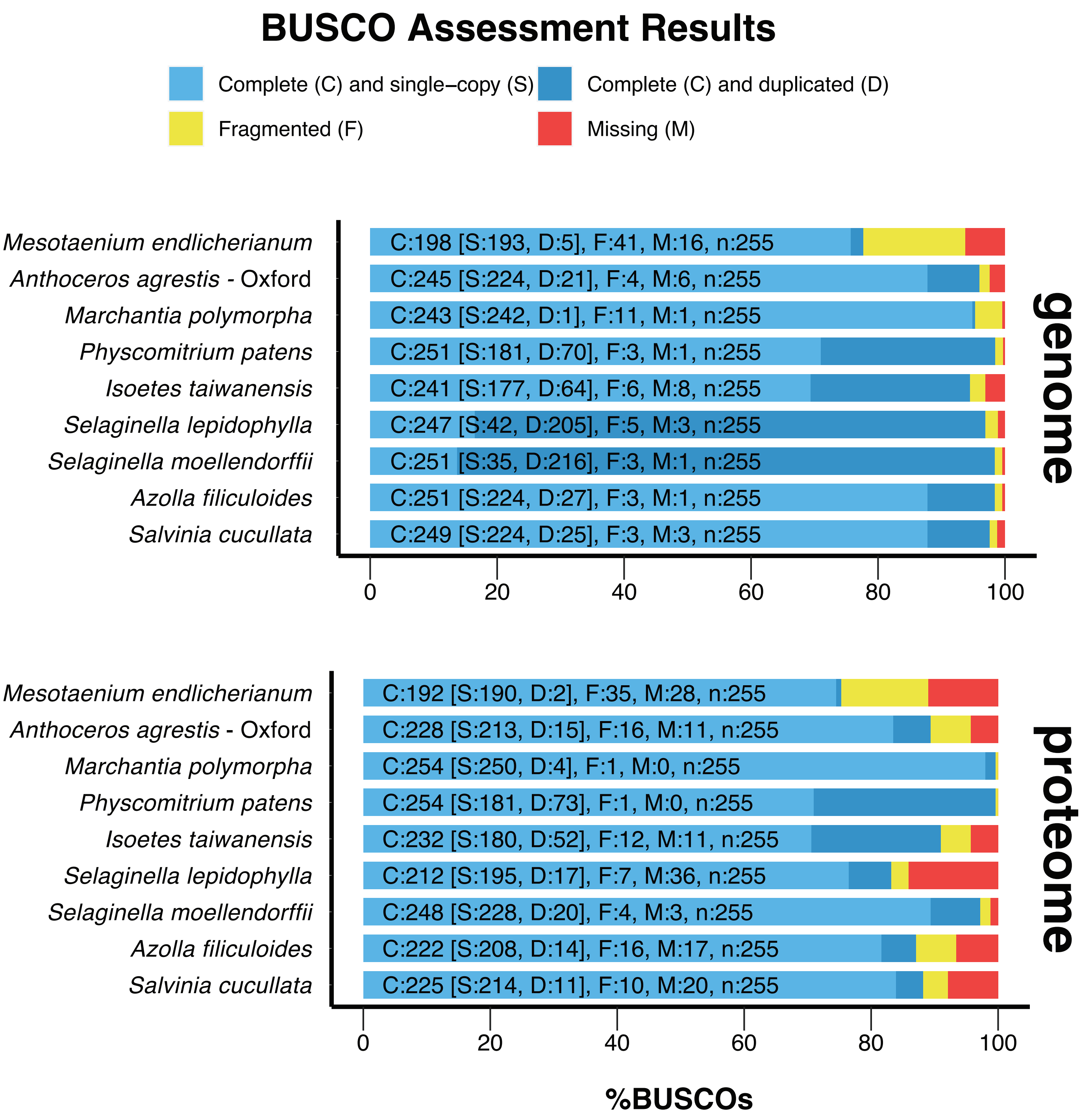

Supplementary Figure 3. Comparison of BUSCO scores for *Isoetes taiwanensis* and other seed-free plant genomes. BUSCO analysis showed that the *I. taiwanensis* genome assembly is comparable to other seed-free plant and charophyte genome assemblies with regard to completeness.

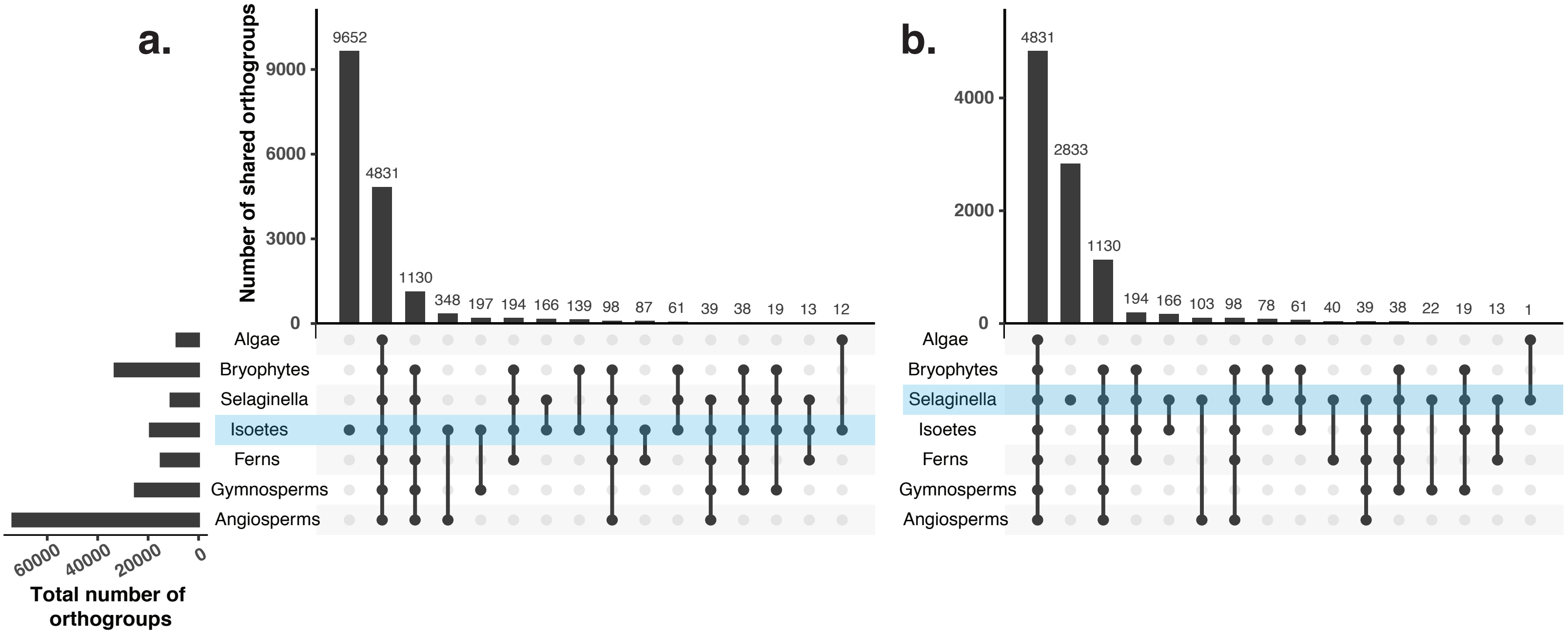

Supplementary Figure 4. Orthogroups in *Isoetes* have greater overlap with seed plants than seed-free taxa. Upset plots show degree of overlap among various plant lineages included in Orthofinder analysis. Each plot focuses on shared orthogroups with a particular group: **a**, *Isoetes taiwanensis*, **b**, *Selaginella moellendorffii*, **c**, ferns, and **d**, bryophytes.

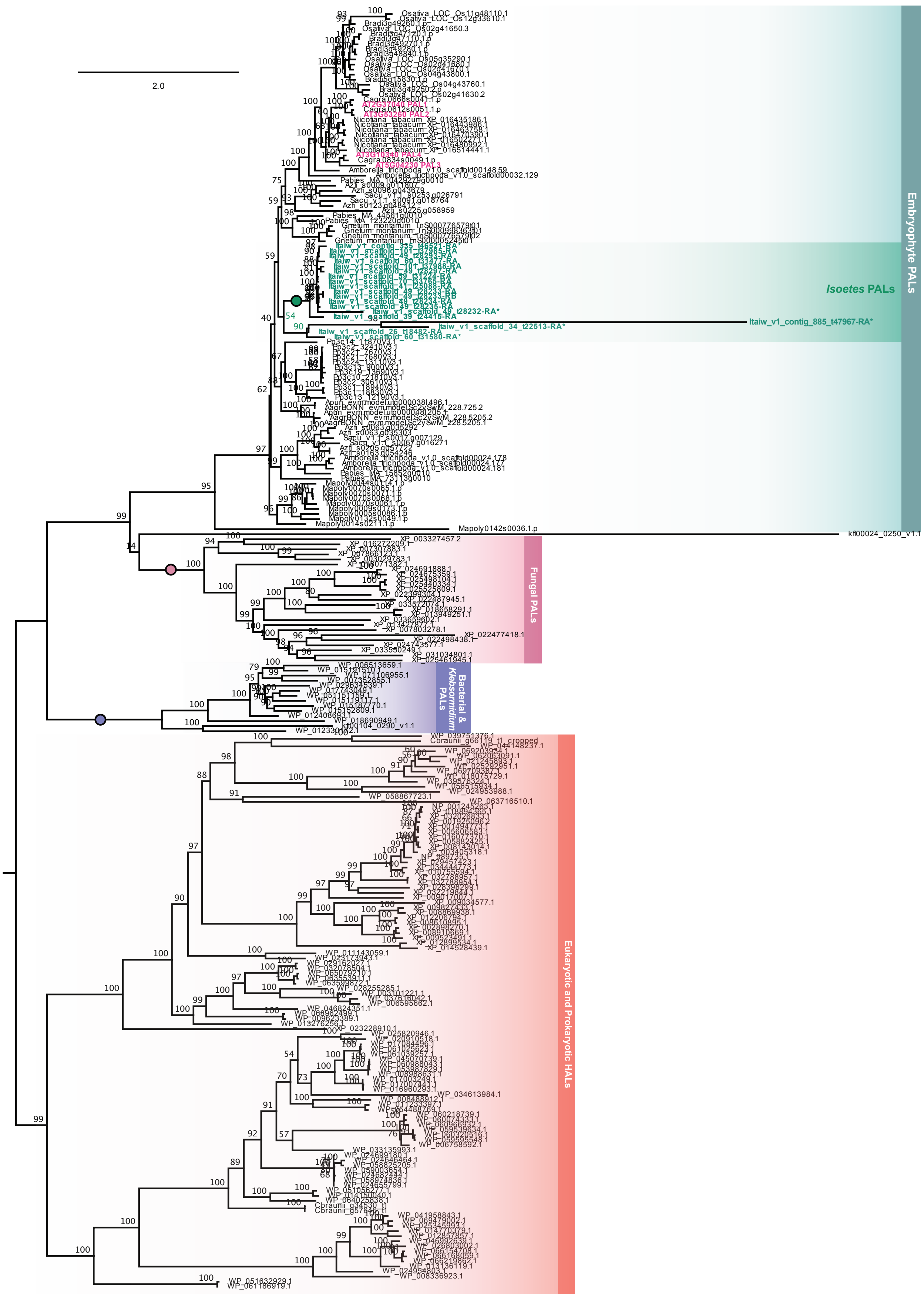

Supplementary Figure 5. Rooted phylogeny of phenylalanine ammonia lyase (PAL) homologs in *Isoetes taiwanensis* and across Chloroplastida. Full bootstrap support for highlighted clades is indicated by a filled circle. Unusually short *I. taiwanensis* sequences in highlighted clades are labeled with an asterisk.

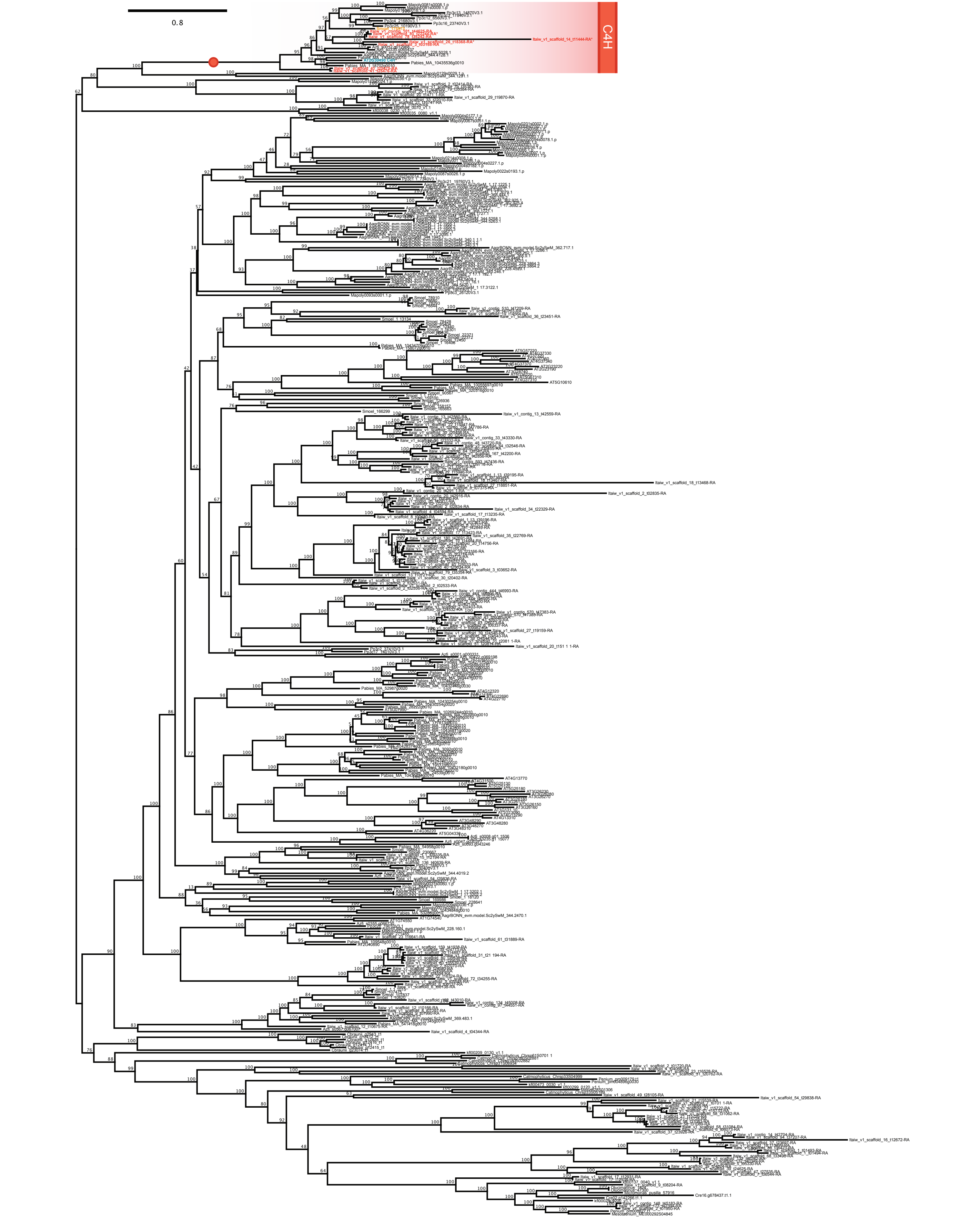

Supplementary Figure 6. Unrooted phylogeny of cinnamate-4-hydroxylase (C4H) homologs in *Isoetes taiwanensis* and across Chloroplastida. Relevant clades are labeled using gradient color; homologues of note are highlighted with colored font (teal for *Arabidopsis*, orange for *Selaginella moellendorffii*, red for *I. taiwanensis*). Full bootstrap support for highlighted clades is indicated by a filled circle. Unusually short *I. taiwanensis* sequences in highlighted clades are labeled with an asterisk.

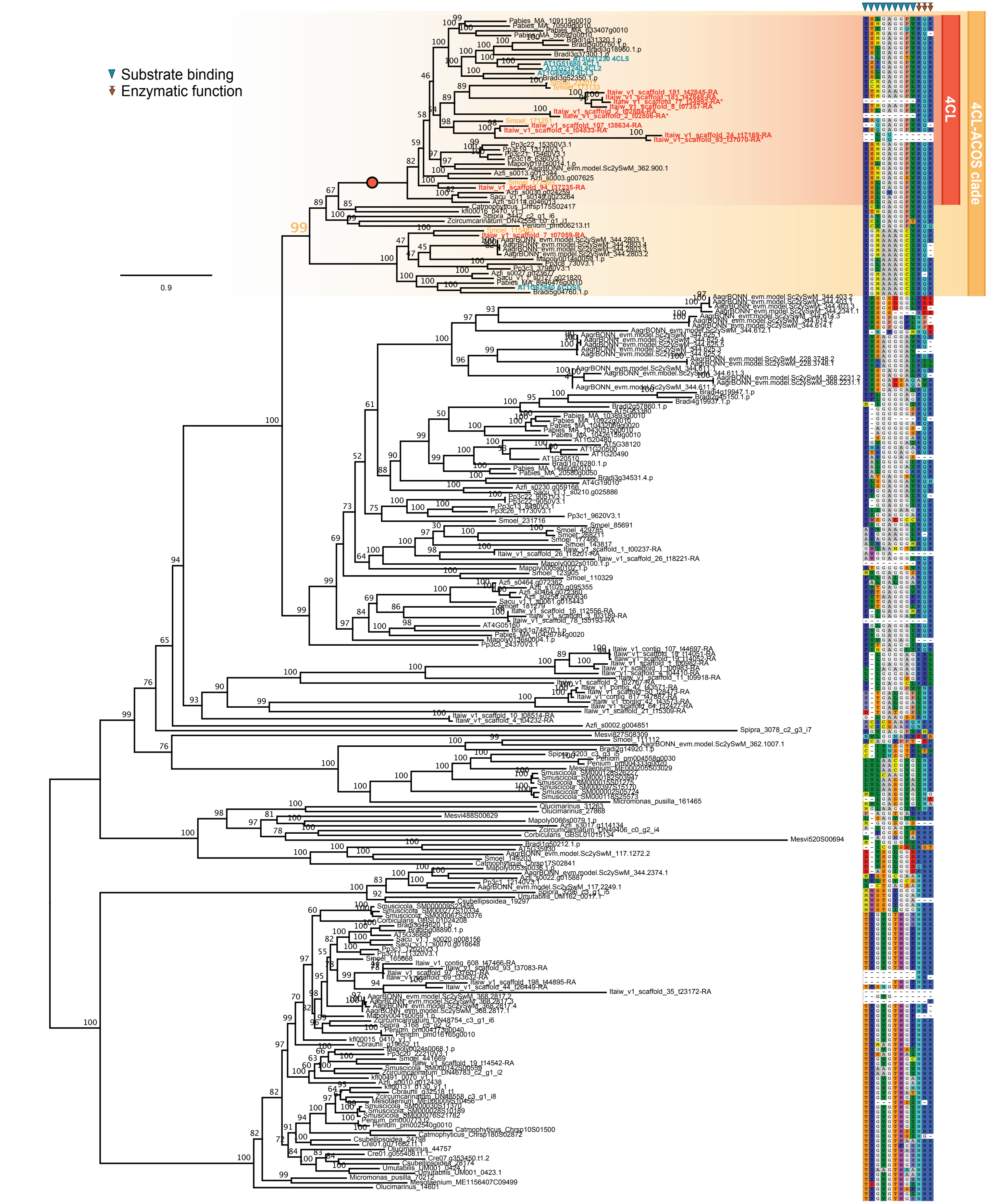

Supplementary Figure 7. Unrooted phylogeny of 4-coumarate CoA ligase (4CL) homologs in *Isoetes taiwanensis* and across Chloroplastida. Relevant clades are labeled using gradient color; homologues of note are highlighted with colored font (teal for *Arabidopsis*, orange for *Selaginella moellendorffii*, red for *I. taiwanensis*). Full bootstrap support for highlighted clades is indicated by a filled circle. To the right of the phylogeny residues relevant to substrate binding and function of canonical 4CL as reported by Hu et al.[40] are shown. Unusually short *I. taiwanensis* sequences in highlighted clades are labeled with an asterisk.

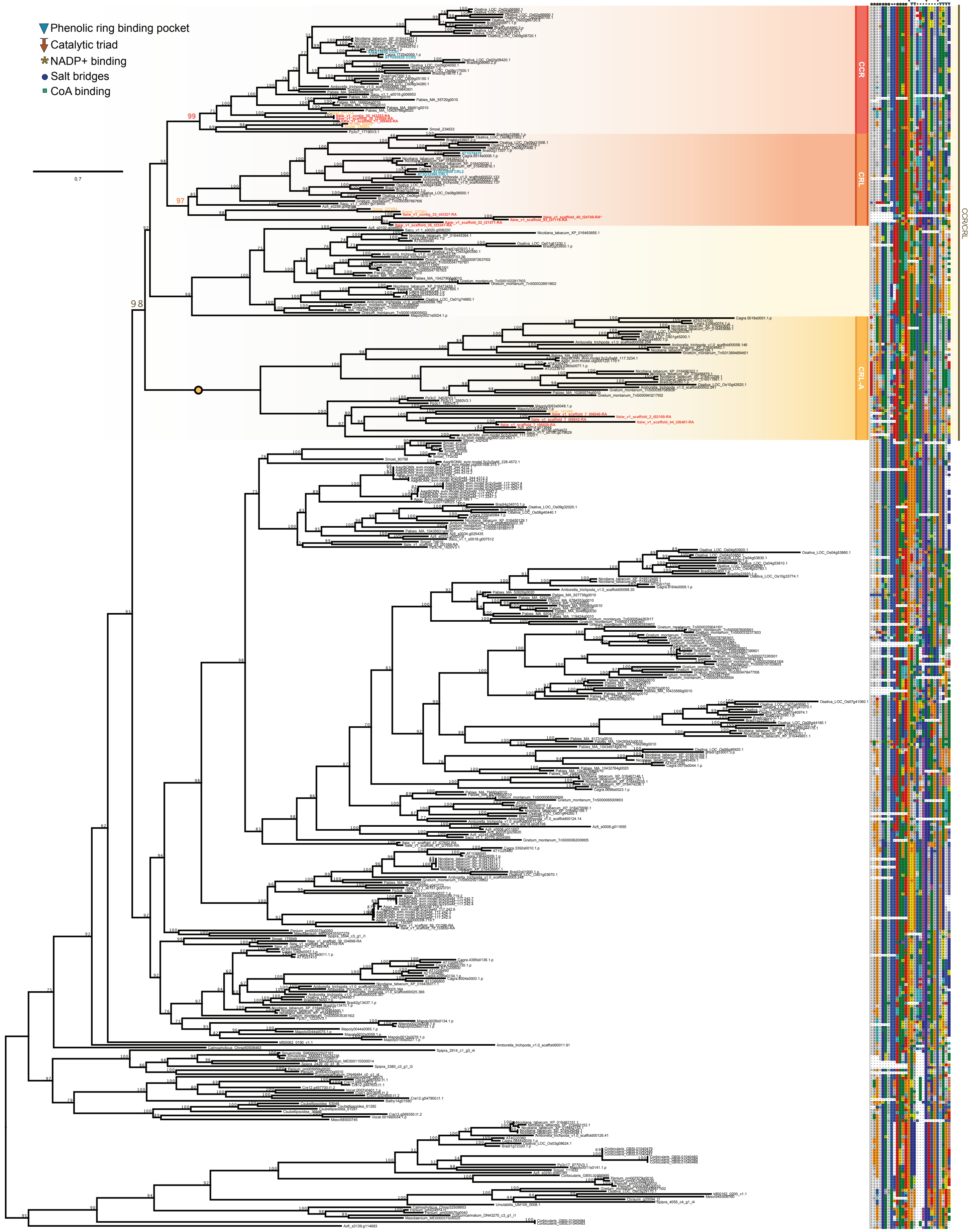

Supplementary Figure 8. Unrooted phylogeny of cinnamoyl CoA reductase (CCR) homologs in *Isoetes taiwanensis* and across Chloroplastida. Relevant clades are labeled using gradient color; homologues of note are highlighted with colored font (teal for *Arabidopsis*, orange for *Selaginella moellendorffii*, red for *I. taiwanensis*). Full bootstrap support for highlighted clades is indicated by a filled circle. To the right of the phylogeny residues relevant to phenolic ring binding pocket, the catalytic triad, NADP+ binding, salt - bridges, and CoA binding of CCR as reported by Pan et al.[41] are shown. Unusually short *I. taiwanensis* sequences in highlighted clades are labeled with an asterisk.

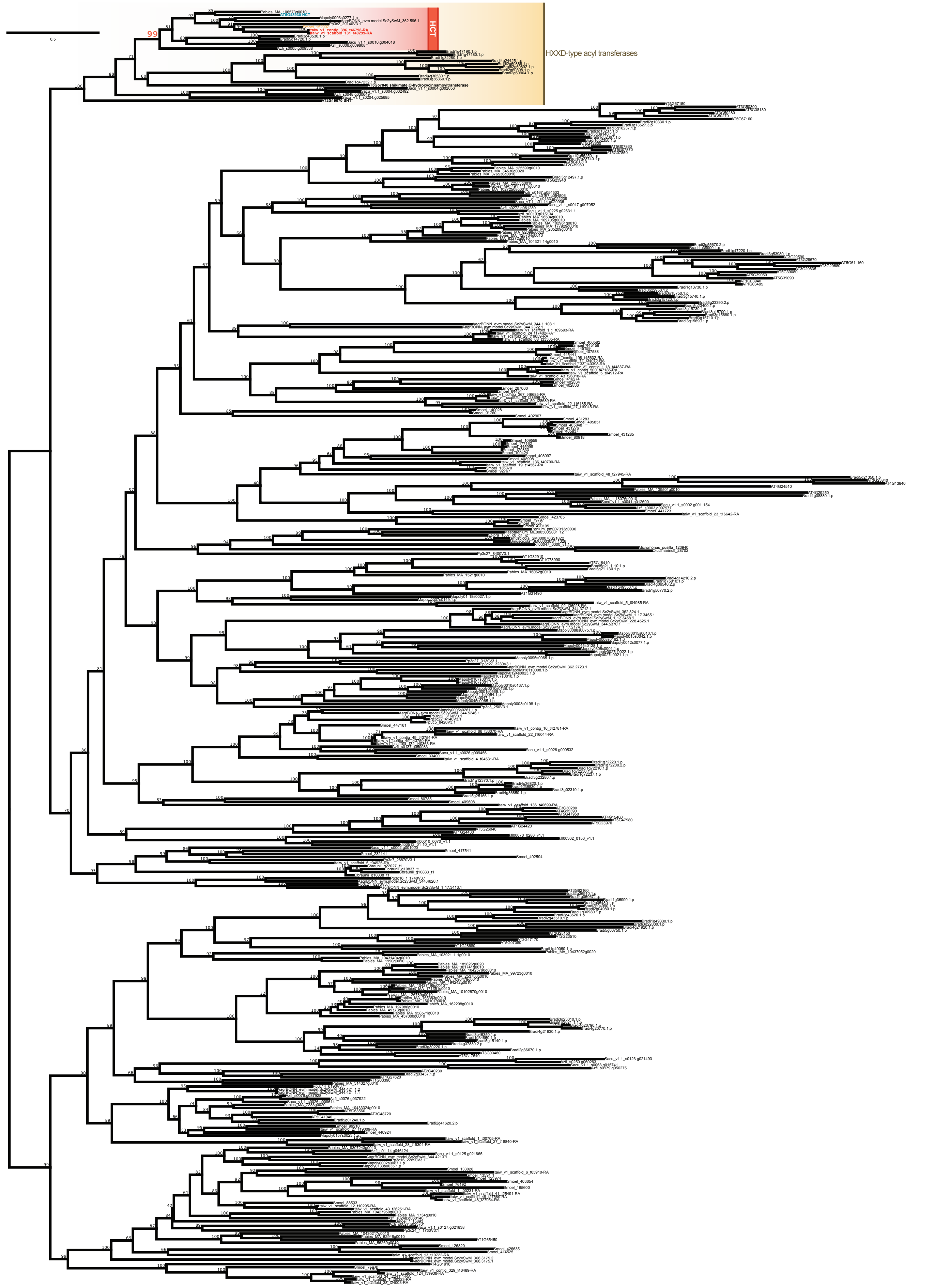

Supplementary Figure 9. Unrooted phylogeny of hydroxycinnamoyl-Coenzyme A shikimae/quinate hydroxycinnamoyltransferase (HCT) homologs in *Isoetes taiwanensis* and across Chloroplastida. Relevant clades are labeled using gradient color; homologues of note are highlighted with colored font (teal for *Arabidopsis*, orange for *Selaginella moellendorffii*, red for *I. taiwanensis*). Full bootstrap support for highlighted clades is indicated by a filled circle.

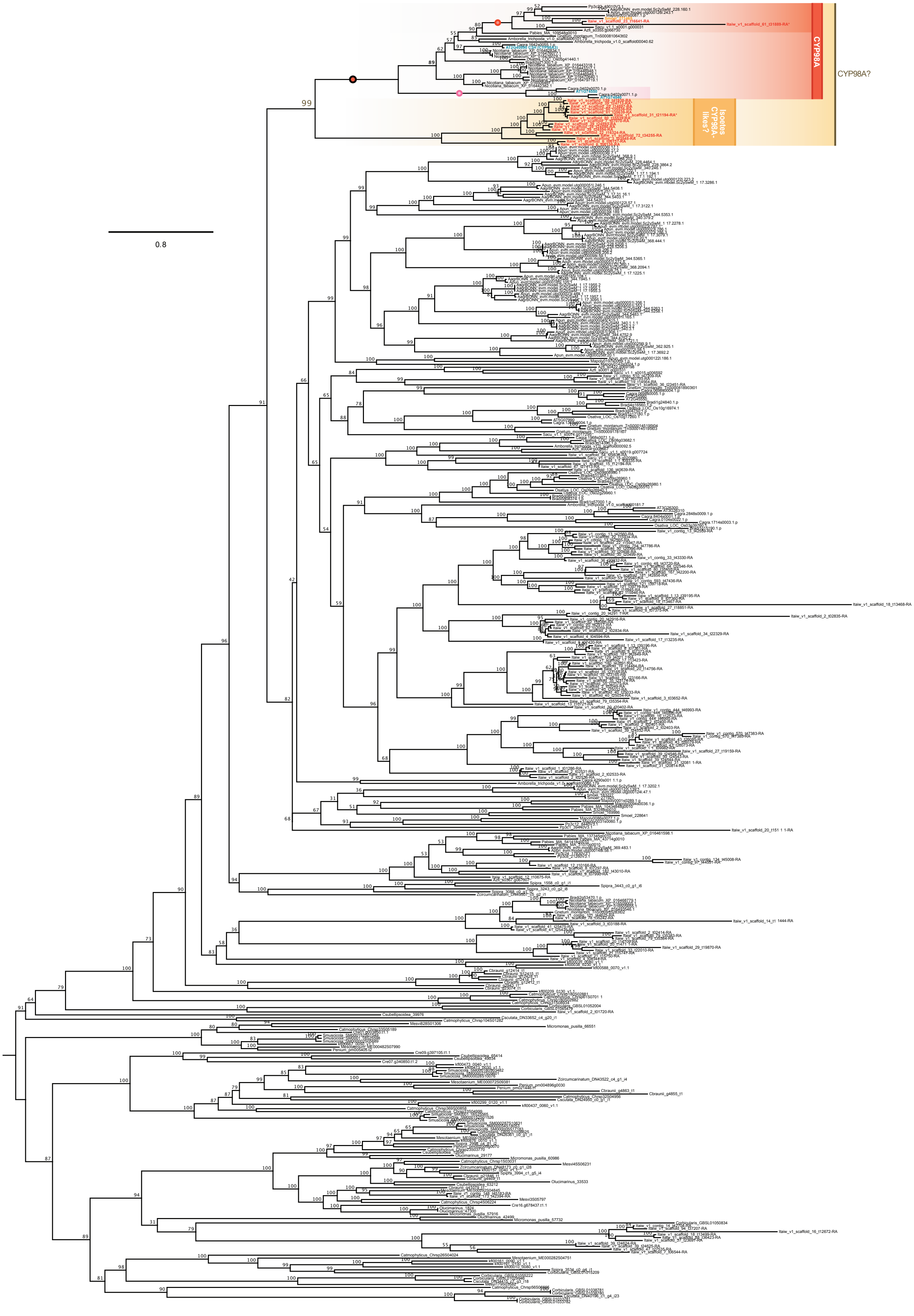

Supplementary Figure 10. Unrooted phylogeny of coumarate 3-hydroxylase (C3H) homologs in *Isoetes taiwanensis* and across Chloroplastida. Relevant clades are labeled using gradient color; homologues of note are highlighted with colored font (teal for *Arabidopsis*, orange for *Selaginella moellendorffii*, red for *I. taiwanensis*). Full bootstrap support for highlighted clades is indicated by a filled circle. Unusually short *I. taiwanensis* sequences in highlighted clades are labeled with an asterisk.

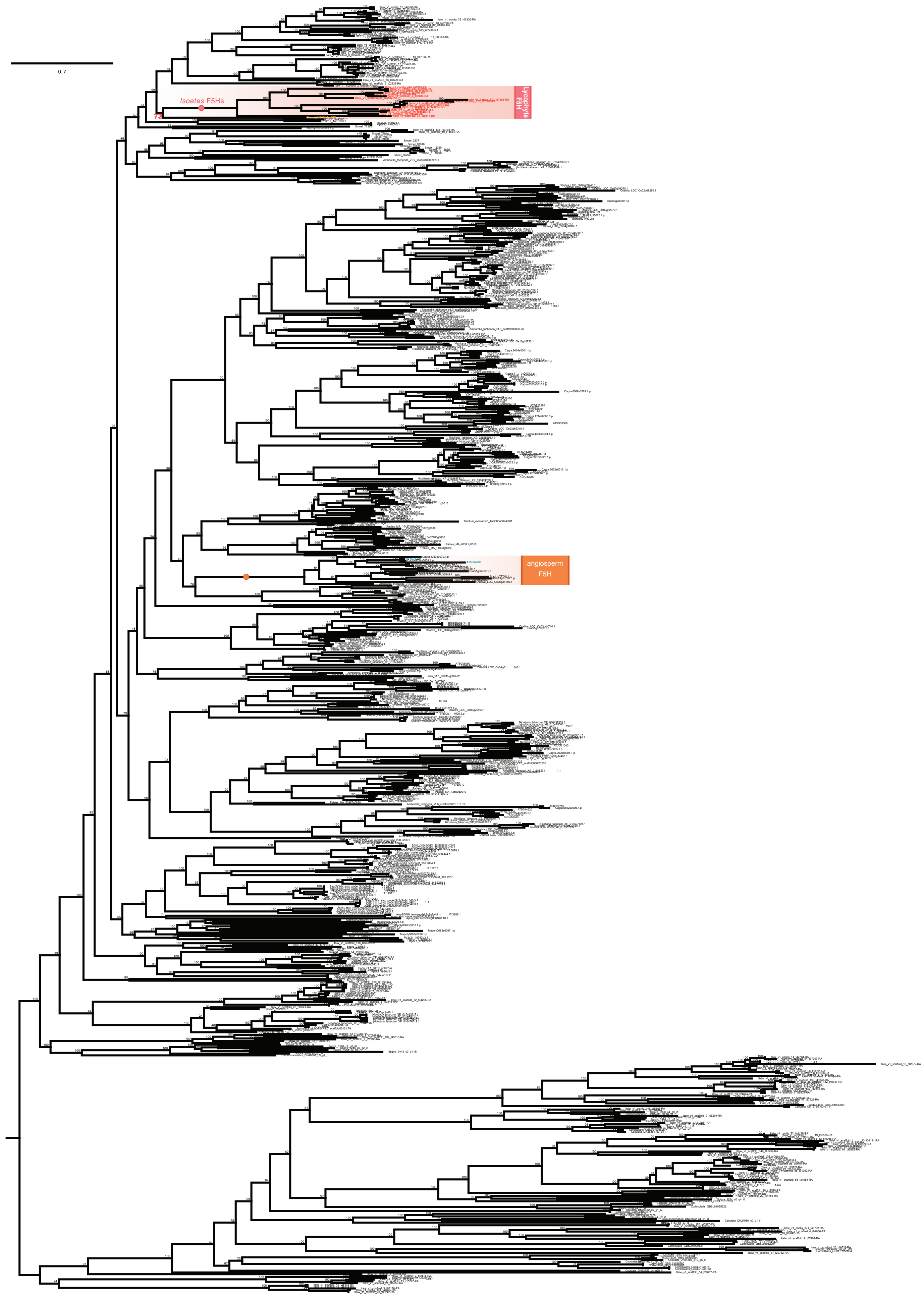

Supplementary Figure 11. Unrooted phylogeny of ferulate 5-hydroxylase (F5H) homologs in *Isoetes taiwanensis* and across Chloroplastida. Relevant clades are labeled using gradient color; homologues of note are highlighted with colored font (teal for *Arabidopsis*, orange for *Selaginella moellendorffii*, red for *I. taiwanensis*). Full bootstrap support for highlighted clades is indicated by a filled circle.

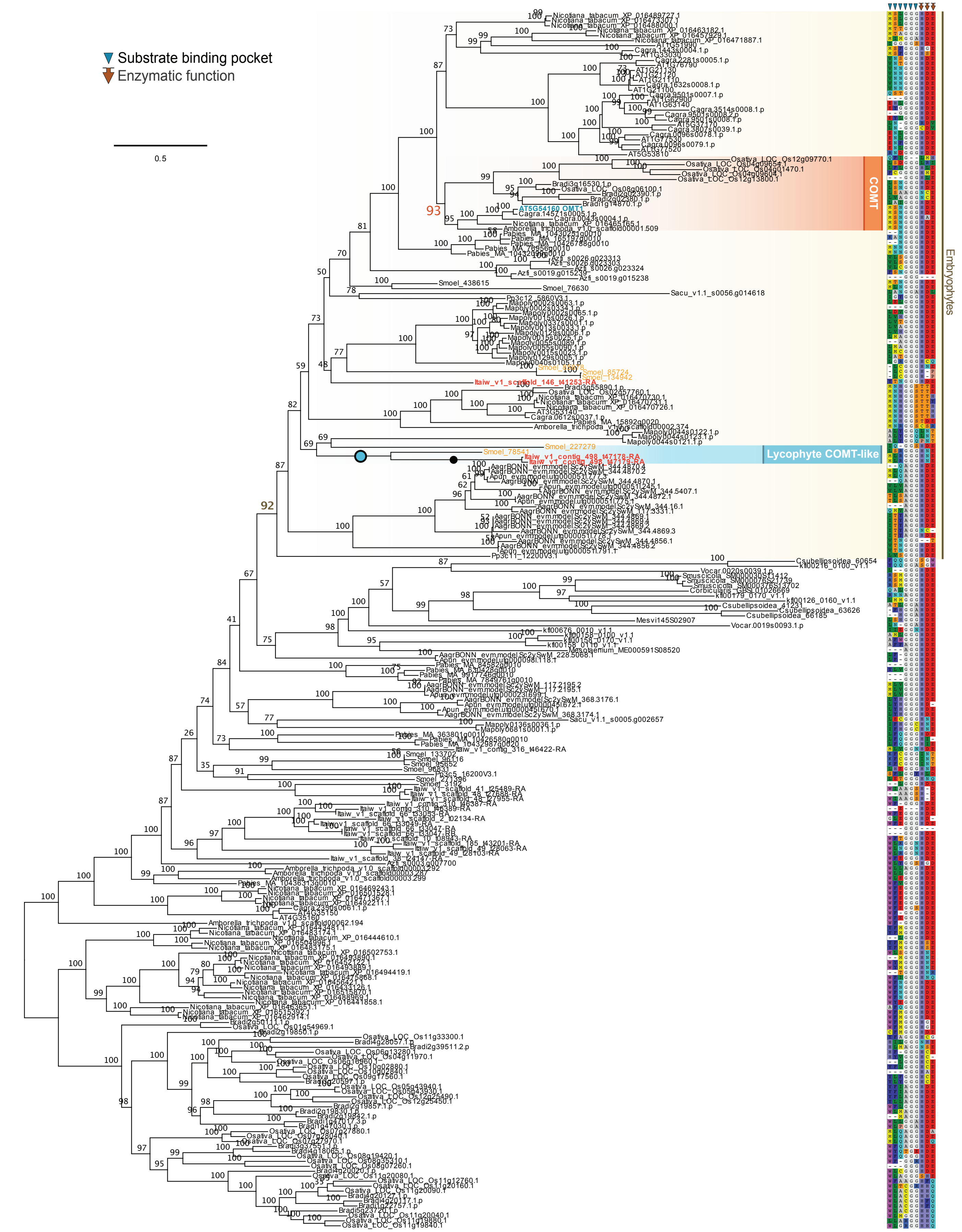

Supplementary Figure 12. Unrooted phylogeny of caffeic acid/5-hydroxyferulic acid O-methyltransferase (COMT) homologs in *Isoetes taiwanensis* and across Chloroplastida. Relevant clades are labeled using gradient color; homologues of note are highlighted with colored font (teal for *Arabidopsis*, orange for *Selaginella moellendorffii*, red for *I. taiwanensis*). Full bootstrap support for highlighted clades is indicated by a filled circle. To the right of the phylogeny residues relevant to substrate binding and function of COMT as reported by Louie et al.[42] are shown.

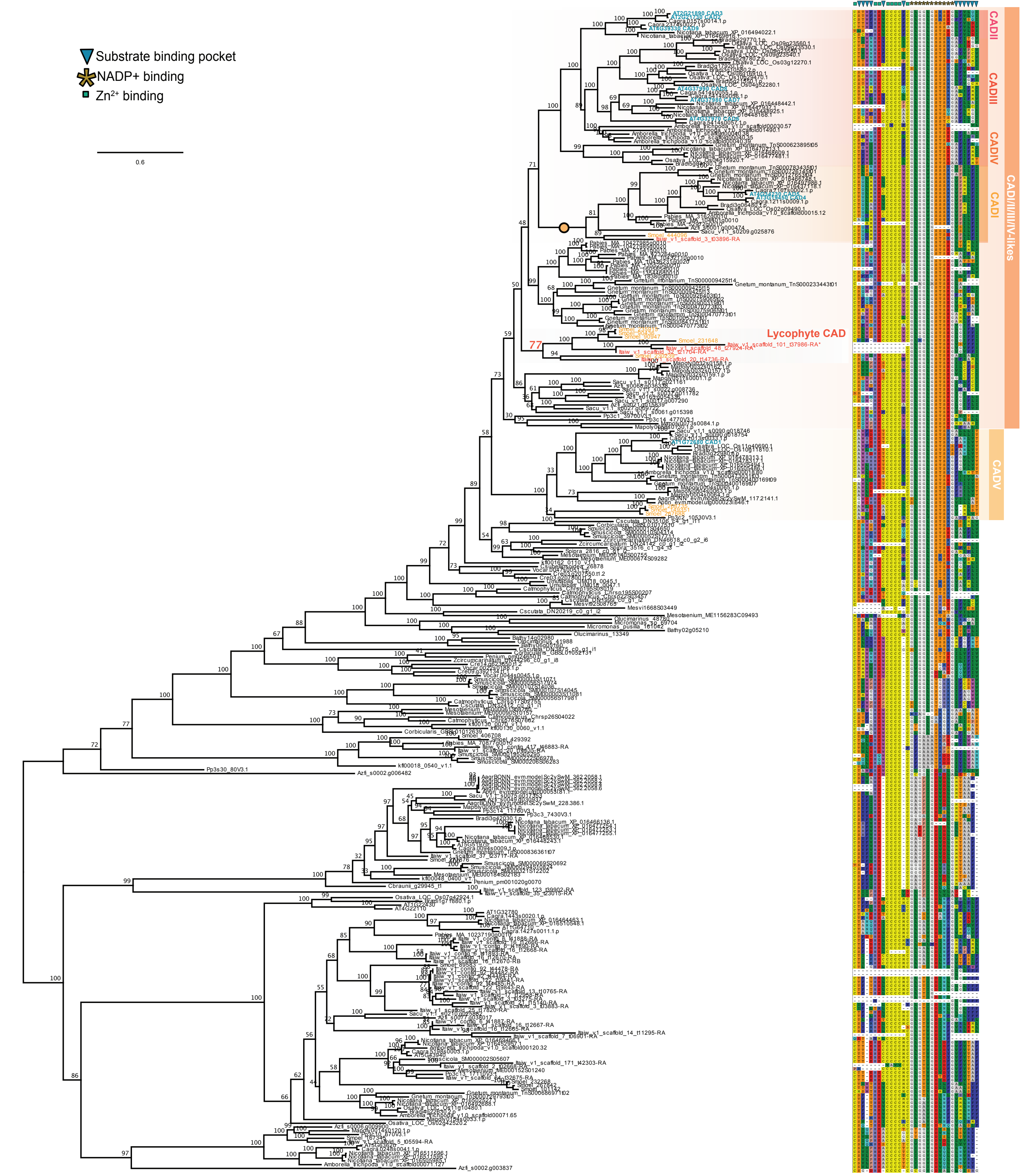

Supplementary Figure 13. Unrooted phylogeny of cinnamyl-alcohol dehydrogenase (CAD) homologs in *Isoetes taiwanensis* and across Chloroplastida. Relevant clades are labeled using gradient color; homologues of note are highlighted with colored font (teal for *Arabidopsis*, orange for *Selaginella moellendorffii*, red for *I. taiwanensis*). Full bootstrap support for highlighted clades is indicated by a filled circle. To the right of the phylogeny residues relevant to substrate binding and function of CAD binding pocket, NADP+- and Zn^{2+} binding according to Youn et al.[43] as reported by Louie et al.[42] are shown. Unusually short *I. taiwanensis* sequences in highlighted clades are labeled with an asterisk.

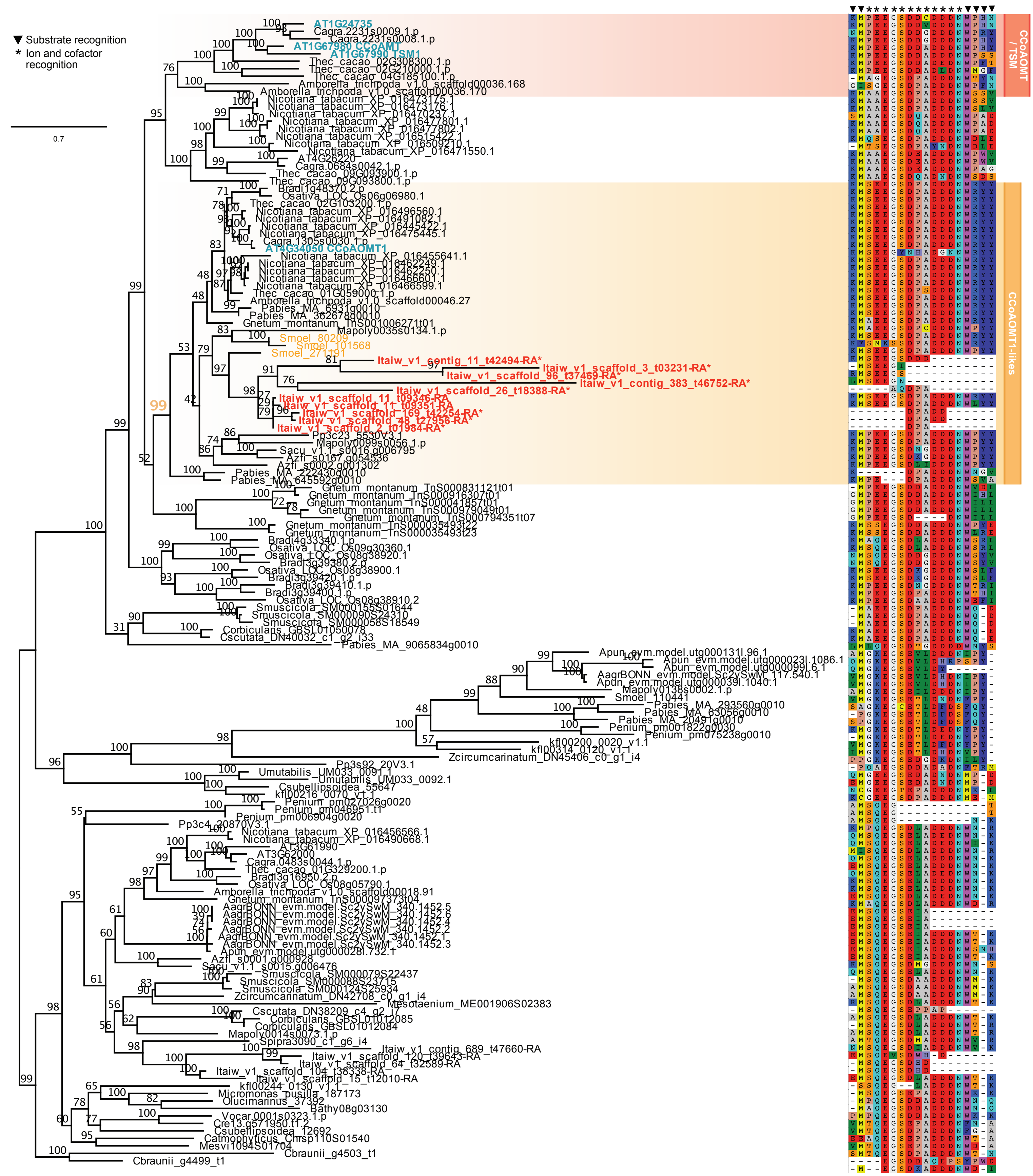

Supplementary Figure 14. Unrooted phylogeny of caffeoyl-CoA O-methyltransferase (CCoAOMT) homologs in *Isoetes taiwanensis* and across Chloroplastida. Relevant clades are labeled using gradient color; homologues of note are highlighted with colored font (teal for *Arabidopsis*, orange for *Selaginella moellendorffii*, red for *I. taiwanensis*). Full bootstrap support for highlighted clades is indicated by a filled circle. To the right of the phylogeny residues relevant to substrate, ion and cofactor recognition by CCoAOMT as reported by Ferrer et al.[44] are shown. Unusually short *I. taiwanensis* sequences in highlighted clades are labeled with an asterisk.

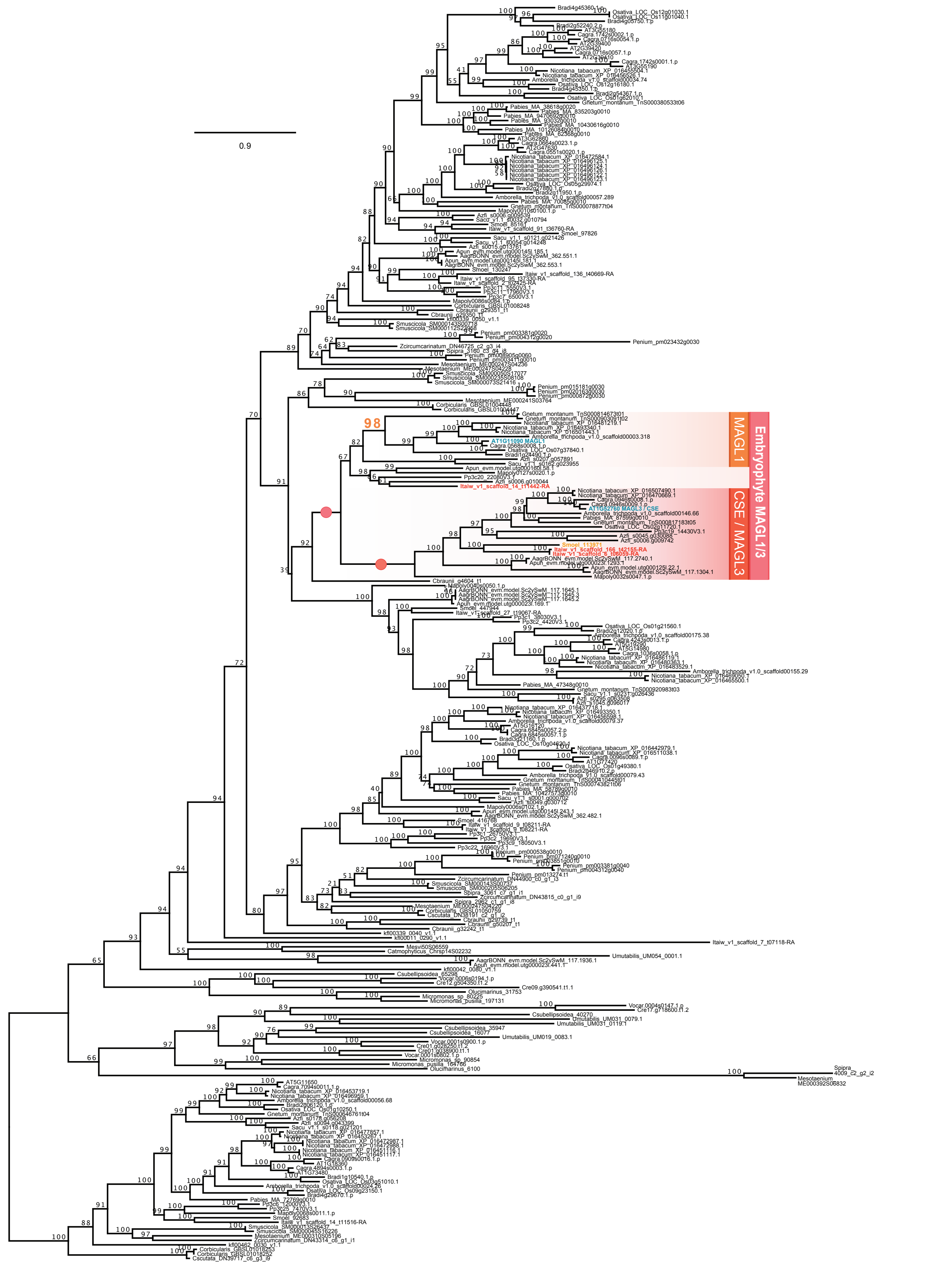

Supplementary Figure 15. Unrooted phylogeny of caffeoyl shikimate esterase (CSE) / monoacylglycerol lipase (MAGL) homologs in *Isoetes taiwanensis* and across Chloroplastida. Relevant clades are labeled using gradient color; homologues of note are highlighted with colored font (teal for *Arabidopsis*, orange for *Selaginella moellendorffii*, red for *I. taiwanensis*). Full bootstrap support for highlighted clades is indicated by a filled circle.

a.

	PAL	C4H	4CL	ACOS5	C3H	CYP98A-likes	HCT	MAGL3/CSE	CCR	CRLA(orange)	CRLB(yellow)	canonical COMT	Lycophyte-type COMT	CCoAMT / CCoAOMT7 / TSM1	CCoAOMT1	canonical F5H	Lycophyte-type F5H	CADI	CADII	CADIII	CADIV	CADV	CAD-likes
Arabidopsis thailana	4	1	4	1	1	0	1	1	2	3	3	1	0	4	1	2	0	2	3	3	0	1	0
Selaginella moellendorffii	0	1	4	1	1	0	1	1	3	2	1	0	2	0	3	0	1	1	0	0	0	3	5
Isoetes taiwanensis	17	8	11	1	2	16	2	2	3	5	5	0	2	0	10	0	16	1	0	0	0	0	4

b.

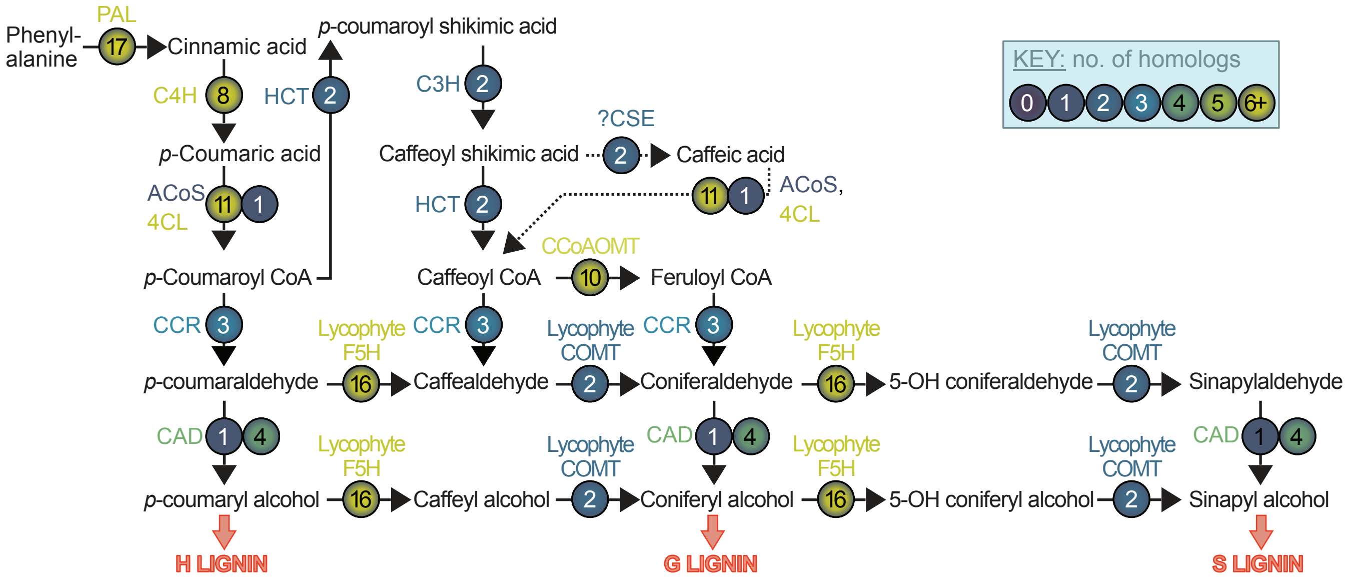

Supplementary Figure 16. Overview of the detected homologues for key enzymes of the phenylpropanoid and lignin biosynthesis pathway in *Isoetes taiwanensis*. a, Phylogenetically detected homologs for key enzymes of the core phenylpropanoid pathway as well as routes towards H, G, and S lignin are distributed across 23 groups. **b,** Putative and simplified biosynthetic routes toward lignin in *I. taiwanensis* as inferred from homologs detected. In both **a** and **b**, the number of detected homologs are shown in gradient

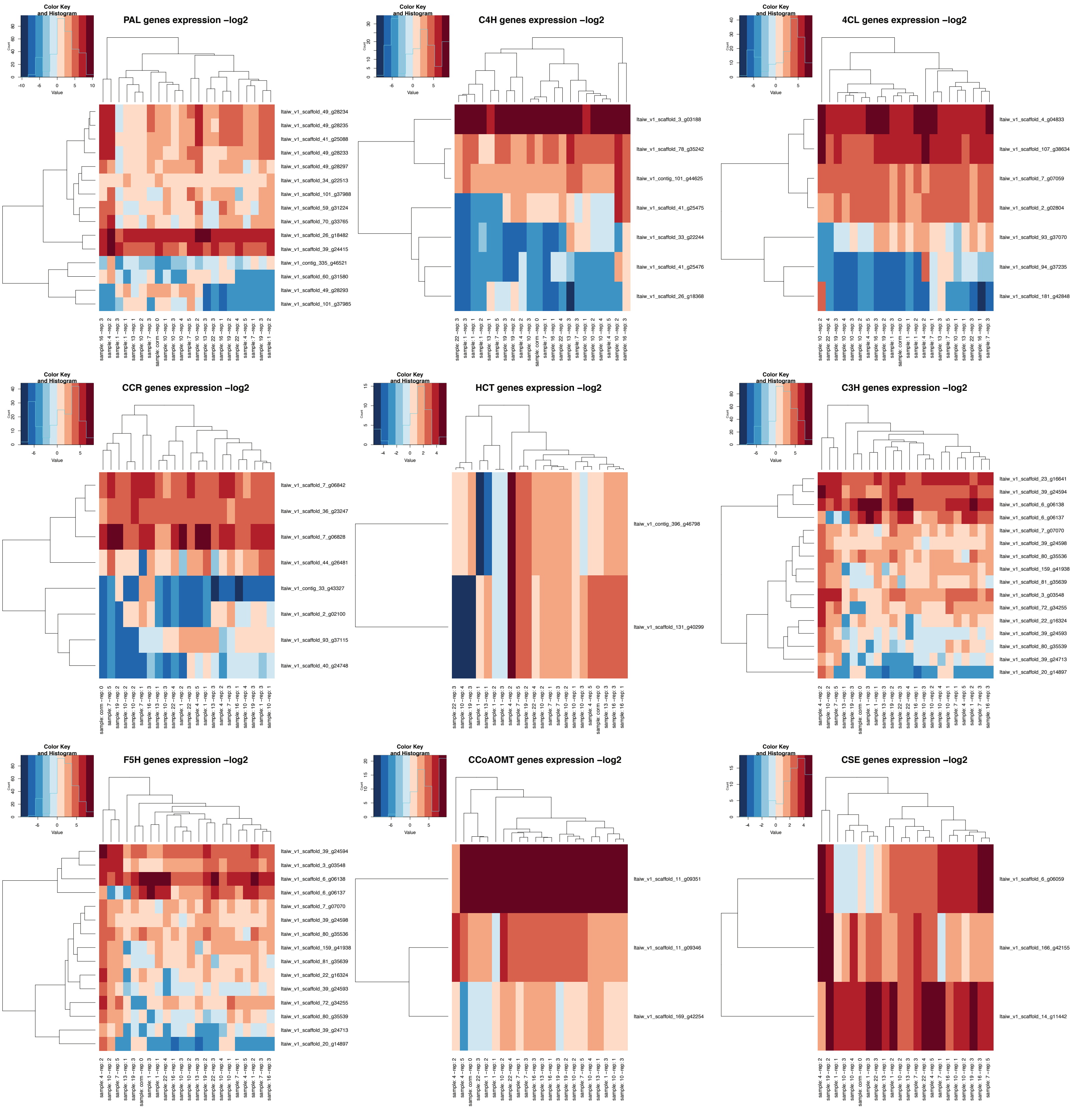

Supplementary Figure 17. Expression of genes in the phenylpropanoid and lignin biosynthesis pathway in *Isoetes taiwanensis* . Note that no COMT homologues and only a single copy of CAD were expressed. The latter is labeled in the phylogeny in Supplemental Figure 13.

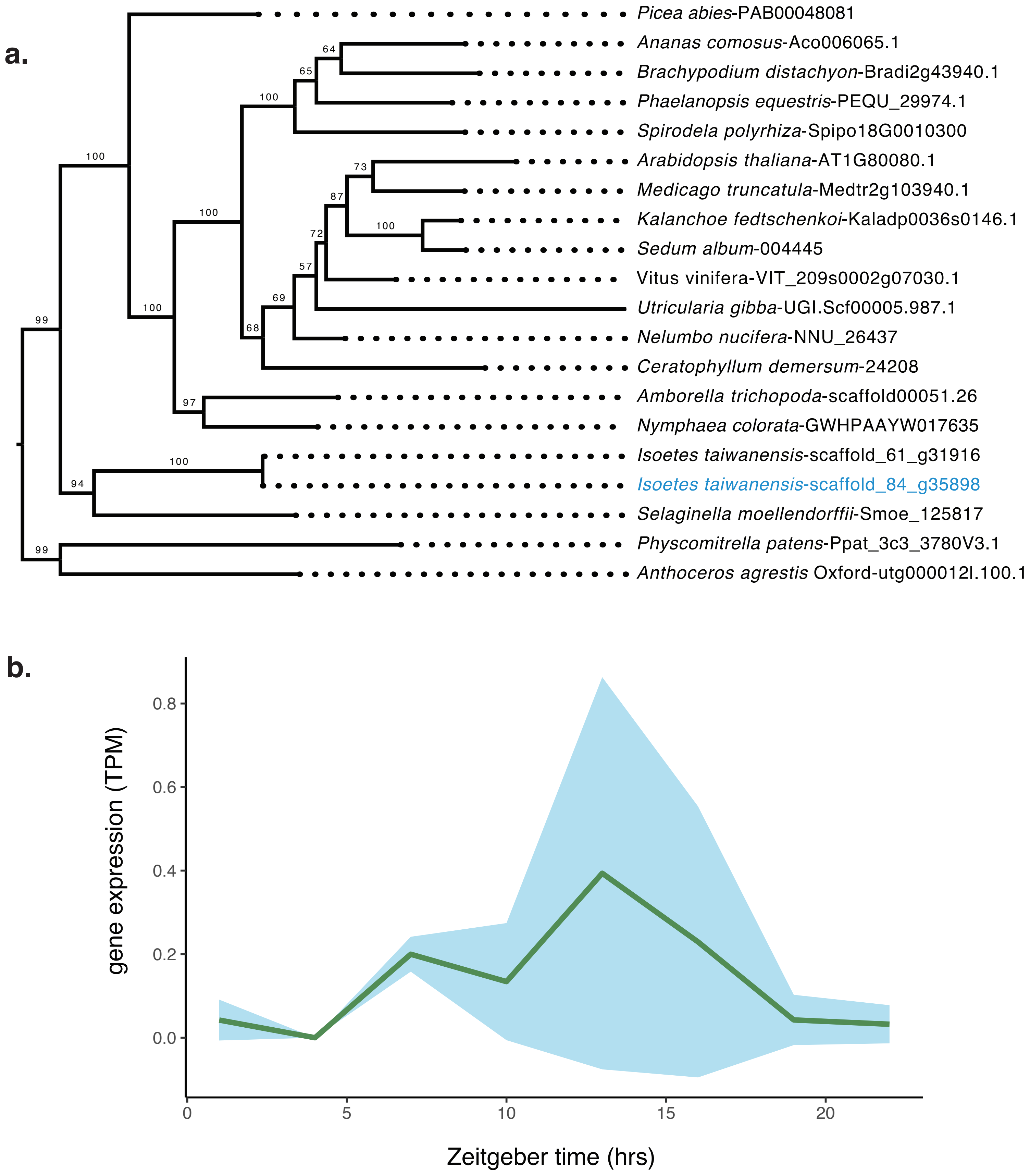

Supplementary Figure 18. Orthologues of *TMM* found in *Isoetes taiwanensis*. a, Phylogenetic placement of two copies of the stomatal development gene *TMM* from *I. taiwanensis* with **b,** normalized expression (TPM) data from one copy expressed in leaf tissue) with a shaded ribbon representing the standard deviation. The copy of *TMM* expressed in leaf tissue is highlighted using blue text in the phylogeny. Source data are provided in Supplementary Data 2.

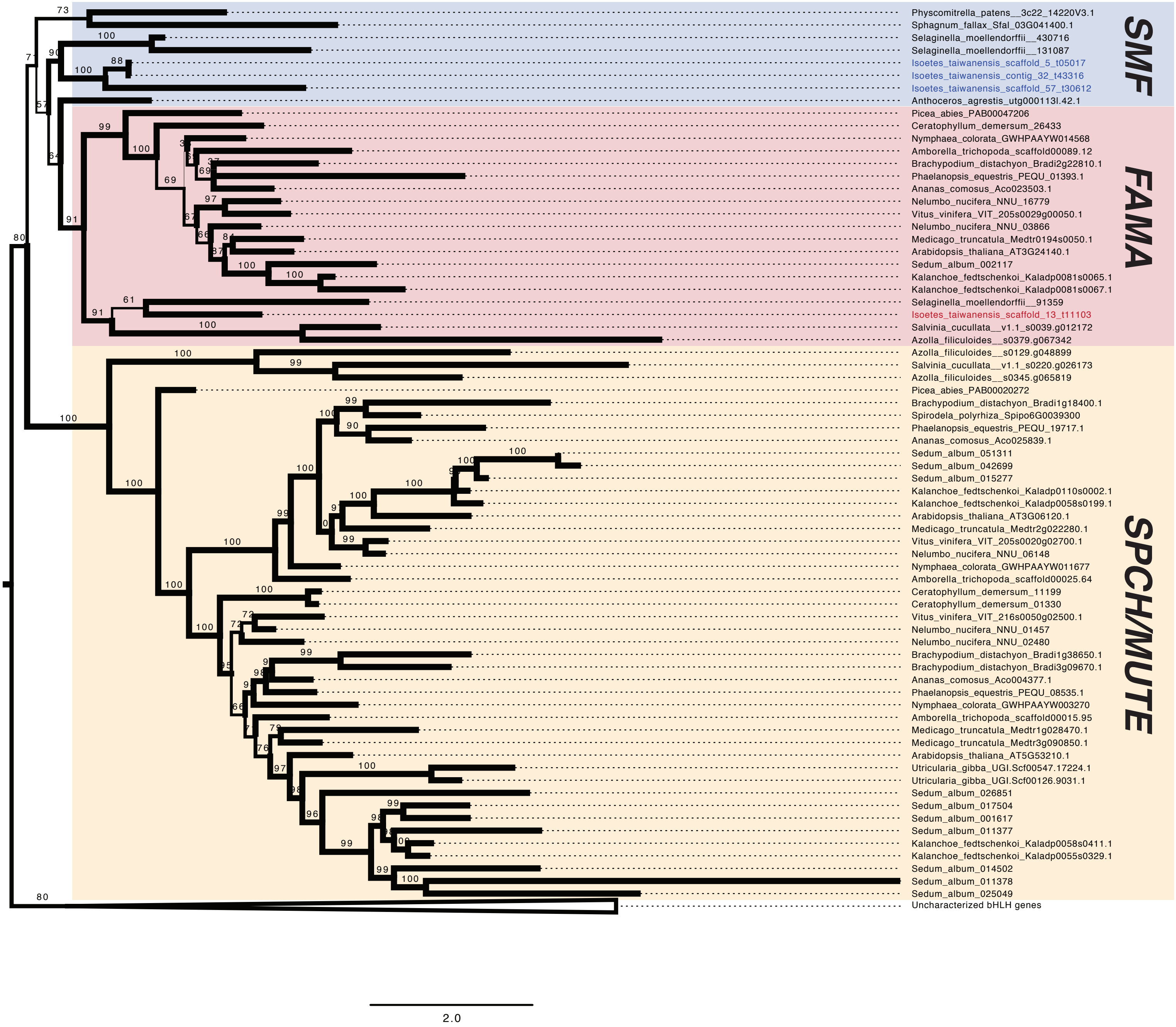

Supplementary Figure 19. ***Isoetes taiwanensis* possesses stomatal development genes in the *SMF/FAMA* clade.** A phylogeny showing the placement of *SMF* and *FAMA* genes found in *I. taiwanensis* relative to other land plants. Branch thickness corresponds to bootstrap support.

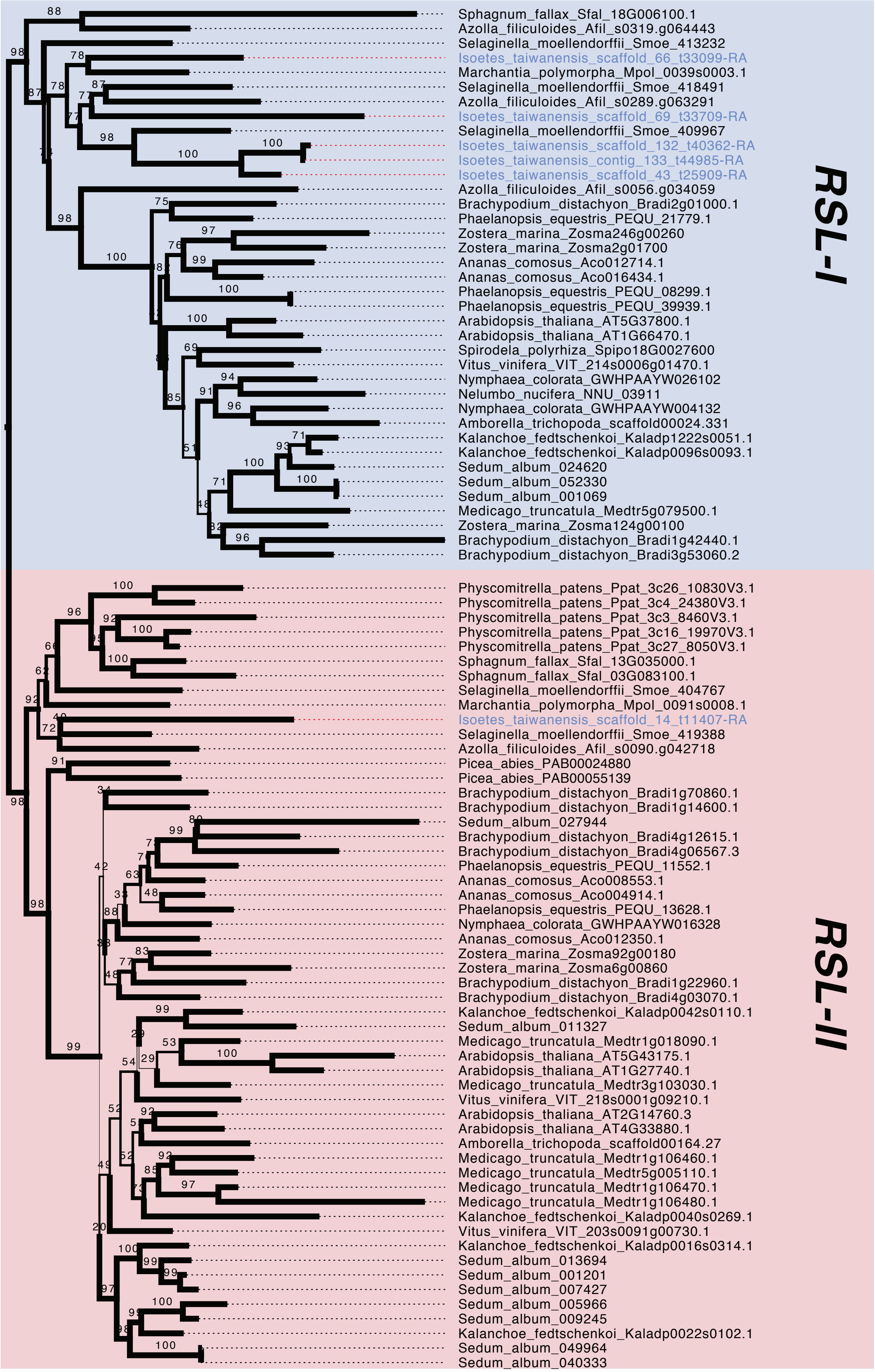

Supplementary Figure 20. ***ROOT HAIR DEFECTIVE SIX-LIKE*** **(*RSL*) genes are present in *Isoetes taiwanensis.*** A phylogeny showing the placement of Class-I and Class-II *RSL* genes found in *I. taiwanensis* relative to other land plants. Branch thickness corresponds to bootstrap support.

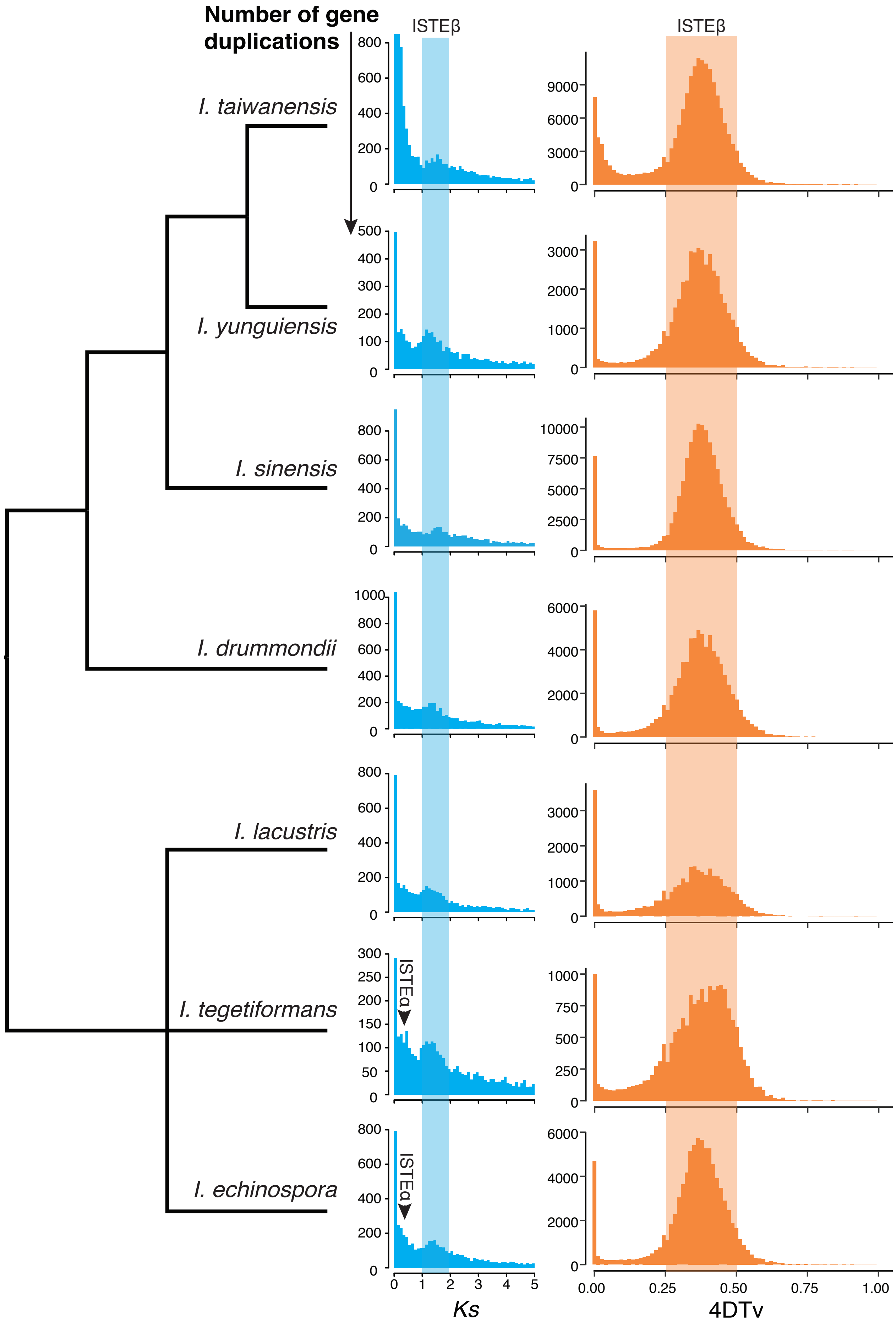

Supplementary Figure 21. A whole genome duplication predates the divergence of two major *Isoetes* clades. Multiple species of *Isoetes* exhibit a *Ks* peak near *Ks* = 1.5-1.8 (blue box), and a 4DTv peak around 0.38 (orange box), corresponding to the ISTEβ event. A *Ks* peak representing a proposed more recent gene duplication identified in *Isoetes tegetiformans* and *Isoetes echinospora* (ISTEα) is indicated by black arrows. The *Ks* and 4DTv plots for *I. taiwanensis* were generated from genomic data while all others were generated from transcriptomic data. Source data are provided as a Source Data file.

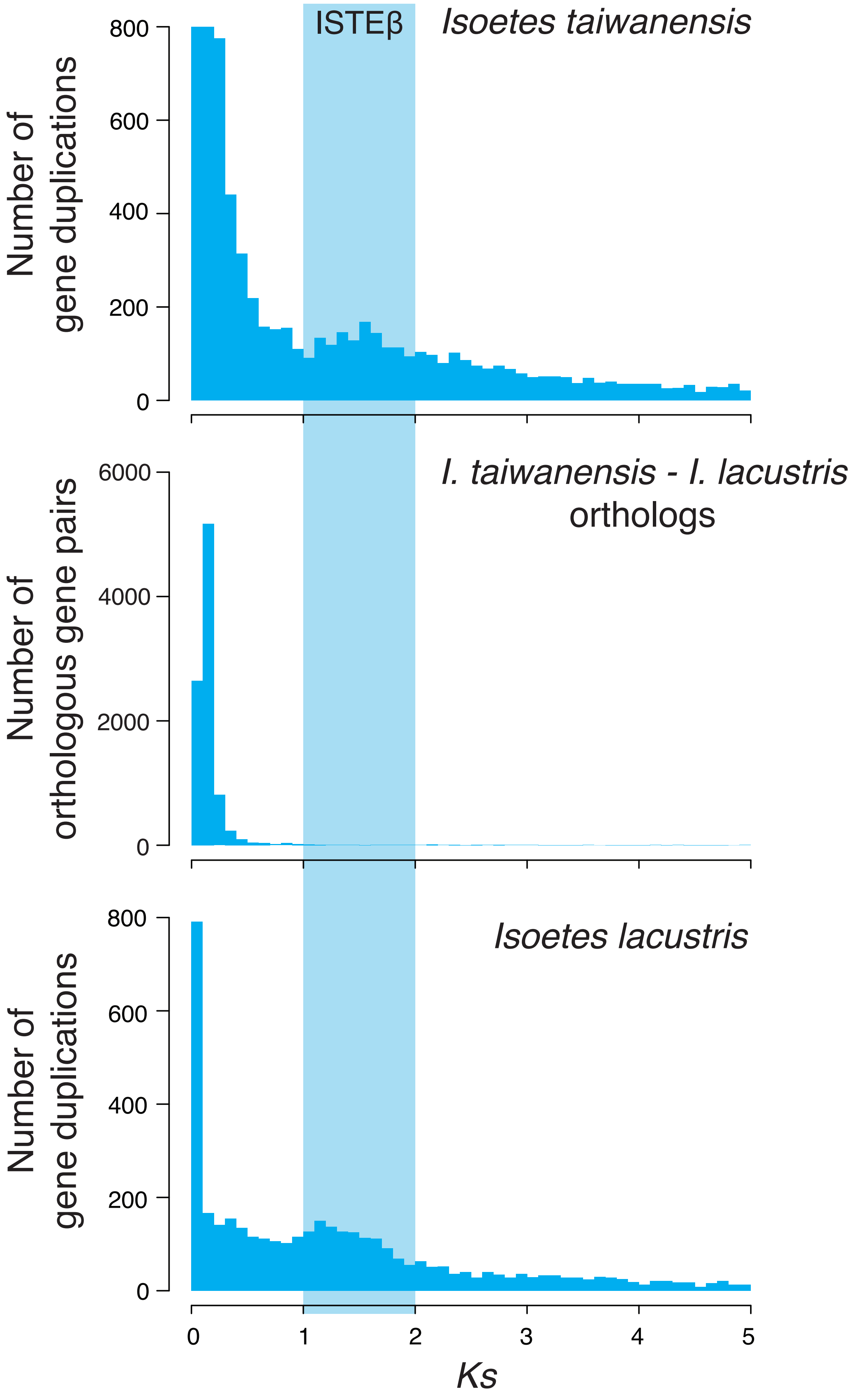

Supplementary Figure 22. A whole genome duplication predates the divergence of two major *Isoetes* clades. Comparison of ortholog divergence (*Ks*) between two distantly related species of *Isoetes* dates an ancient WGD (ISTEβ; blue box) to before the divergence of their respective clades. The *Ks* plot for *I. taiwanensis* was generated from genomic data and the initial peak truncated to highlight ISTEβ peak. The *Ks* plot for *I. lacustris* was generated from transcriptomic data. Source data are provided as a Source Data File.

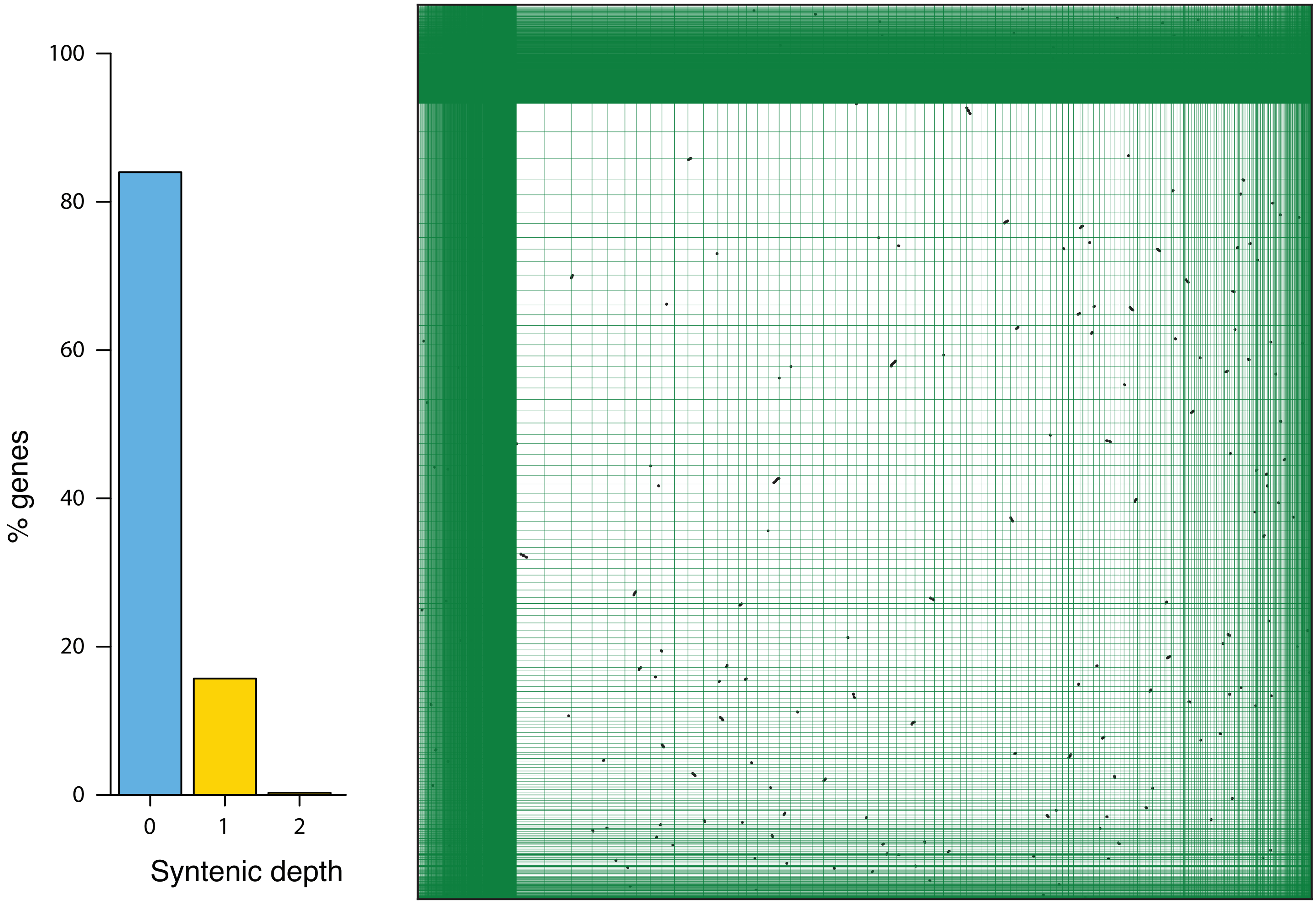

Supplementary Figure 23. ***Isoetes taiwanensis*** **self synteny plot.** Collinearity analysis identified 6,196 genes in 107 collinear blocks. The bar graph at the left shows the proportion of syntenic depth throughout the genome. Source data are provided as a Source Data file.

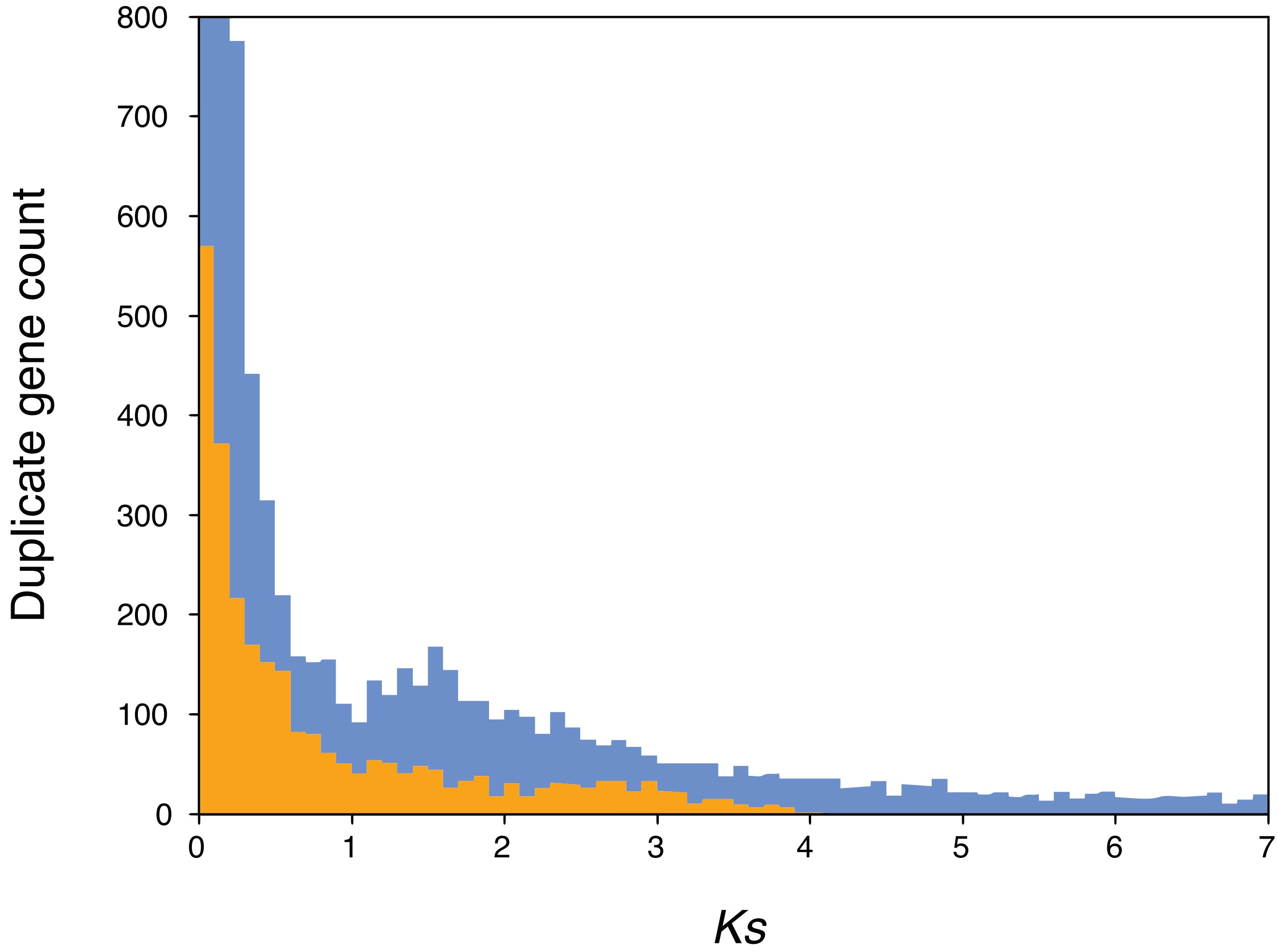

Supplementary Figure 24. *Ks* distribution of syntenic gene pairs differs from that of the whole genome. A peak in the genome-wide *Ks* distribution (blue) around 1.8 was not evident when *Ks* analysis was restricted to syntenic gene pairs (orange). Source data are provided as a Source Data file.

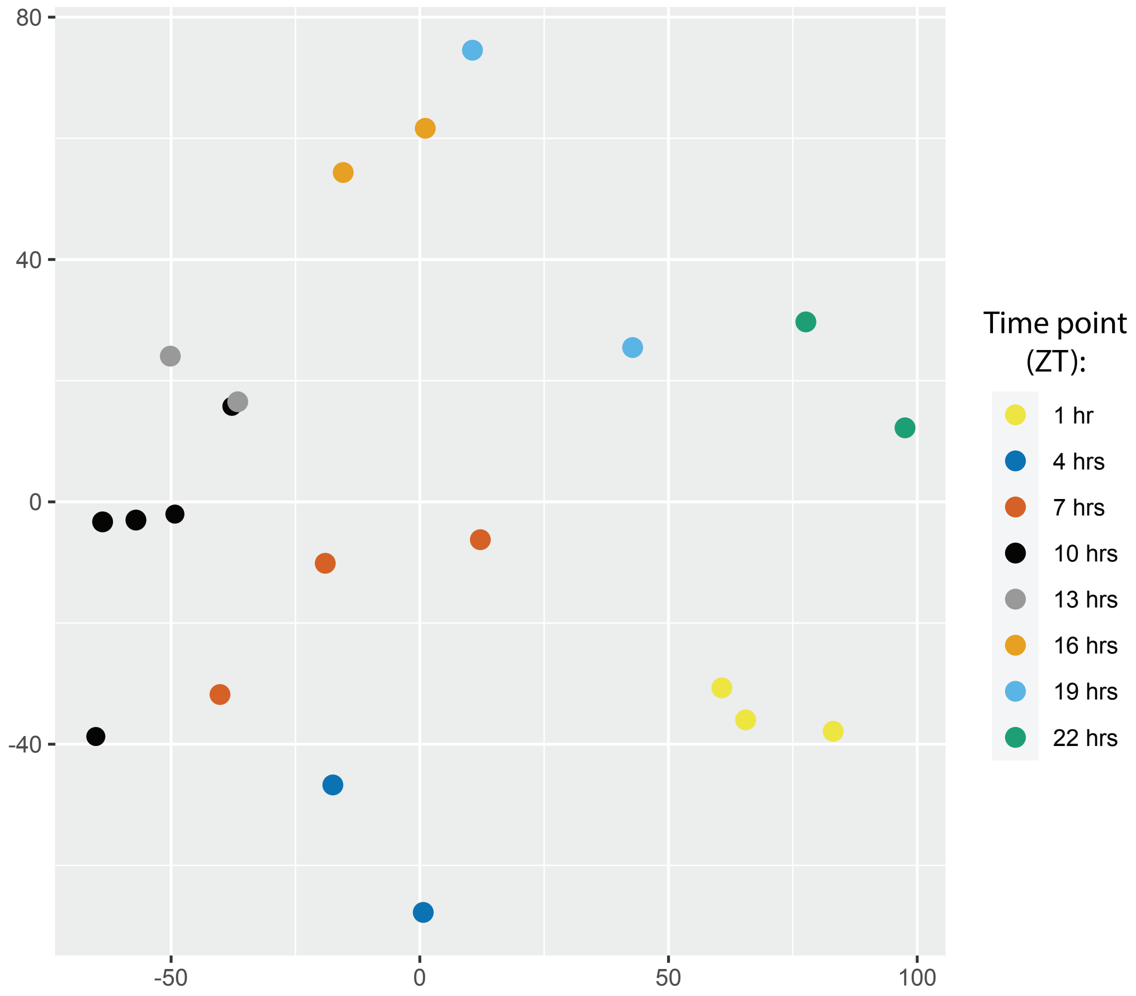

Supplementary Figure 25. RNA-seq samples used for gene expression analyses cluster together by time point. A Multidimensional scaling plot of RNA-seq data shows clustering by time point in a clockwise manner following the removal of outliers. Source data are provided in Supplementary Data 2.

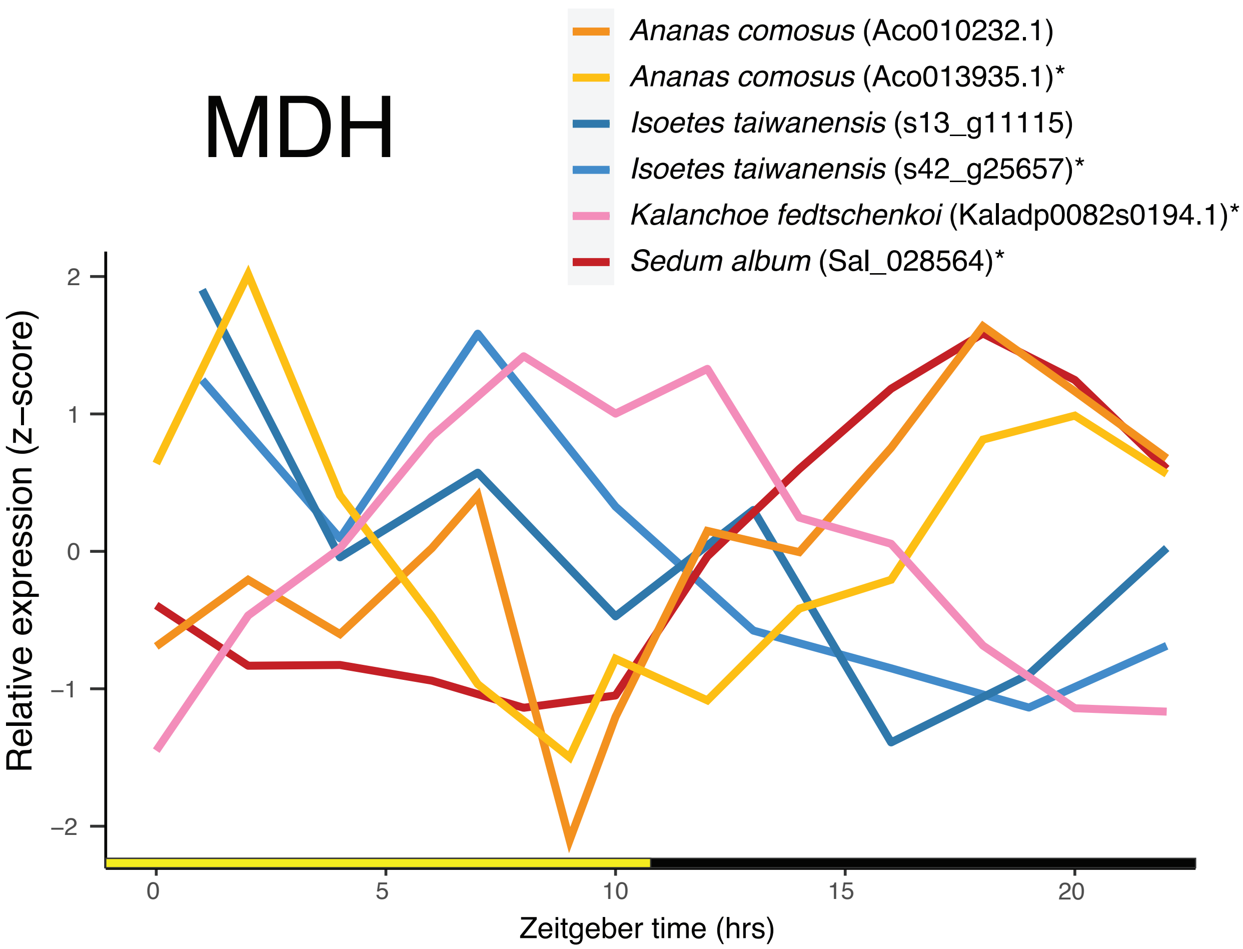

Supplementary Figure 26. Comparison of cycling copies of MDH across CAM species. Plot shows relative gene expression (z-score of TPM normalized RNAseq data) for all MDH copies that are known to cycle in terrestrial CAM plants as well as two copies from *Isoetestaiwanensis*. Orthologous genes are marked with an asterisk (*). Source data are provided for *I. taiwanensis* in Supplementary Data 2 and for other taxa in a Source Data file.

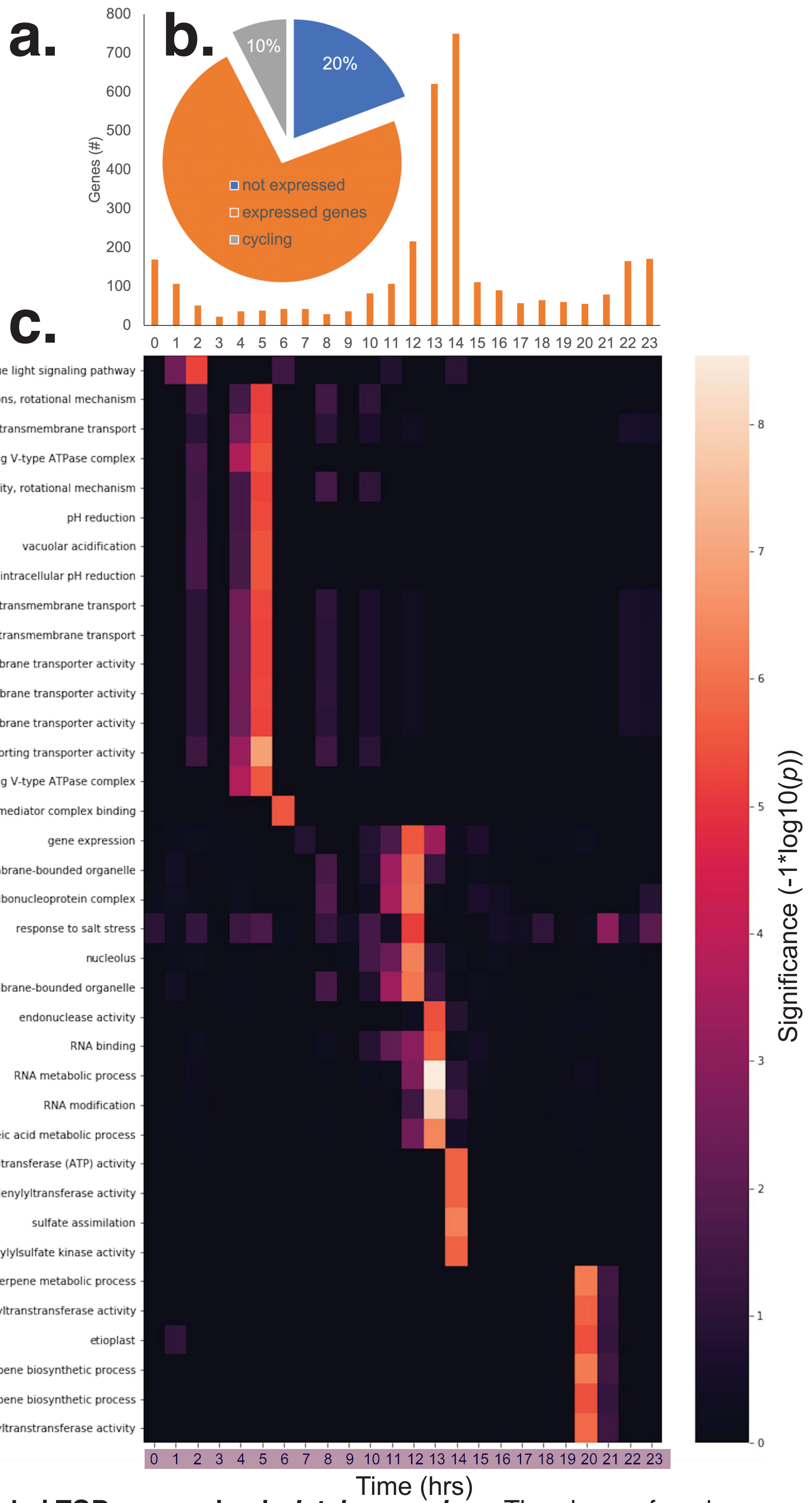

Supplementary Figure 27. Global TOD expression in *I. taiwanensis*. a, The phase of peak expression of the cycling genes as called by HAYSTACK. **b,** The number of cycling genes (grey; 10% of expressed genes) compared to the expressed (orange) and non-expressed (blue). **c,** Enriched GO terms at each time point. GO terms are plotted by significance (-1 * log10(p)) with highly significant (white) and non-significant (black) values for each time over the day to highlight the TOD specificity. Source data are provided in Supplementary Data 3.

a.

	LDHC	LLHC	LDHH_ST	LDHH_SM	long	short	LL_LDHC	LL_LLHC	LL12_LDHH	LL23_LDHH	DD_DDHC	Isoetes
0	115	124	115	74	54	56	68	79	47	26	46	60
1	103	104	93	77	63	71	40	55	70	32	42	41
2	86	78	40	64	66	50	42	42	60	35	21	19
3	114	79	62	75	123	185	35	53	66	66	28	7
4	109	81	58	84	95	181	24	32	50	70	32	19
5	159	105	84	99	150	318	43	39	75	93	16	16
6	154	114	70	60	97	341	60	35	51	91	6	17
7	145	211	78	54	84	277	66	59	56	113	7	25
8	319	538	315	145	185	339	127	136	63	203	14	14
9	52	162	101	19	52	35	36	56	20	56	4	29
10	188	280	191	87	271	187	114	132	43	112	8	53
11	186	187	236	124	103	113	66	74	44	100	12	105
12	150	103	180	110	96	115	69	56	38	97	17	137
13	109	91	150	129	91	122	79	53	61	85	23	197
14	81	63	130	86	97	85	71	27	53	127	16	188
15	140	72	76	76	151	103	101	55	70	128	14	43
16	163	78	72	40	99	133	97	40	79	97	11	41
17	217	102	85	68	91	222	98	44	87	114	11	31
18	260	170	88	60	56	572	102	60	83	112	11	32
19	284	323	109	96	66	234	63	68	65	88	9	21
20	243	372	193	171	88	142	53	79	47	80	24	18
21	245	357	175	211	196	72	67	95	47	51	12	31
22	205	265	127	126	389	57	68	95	56	56	8	67
23	150	197	241	224	174	82	88	117	55	39	43	69
sum	3,977	4,256	3,069	2,359	2,937	4,092	1,677	1,581	1,386	2,071	435	1,280
RHB (%)	49	53	38	29	36	51	21	20	17	26	5	16
Total (%)	38	41	28	22	29	39	15	16	14	20	4	10

b.

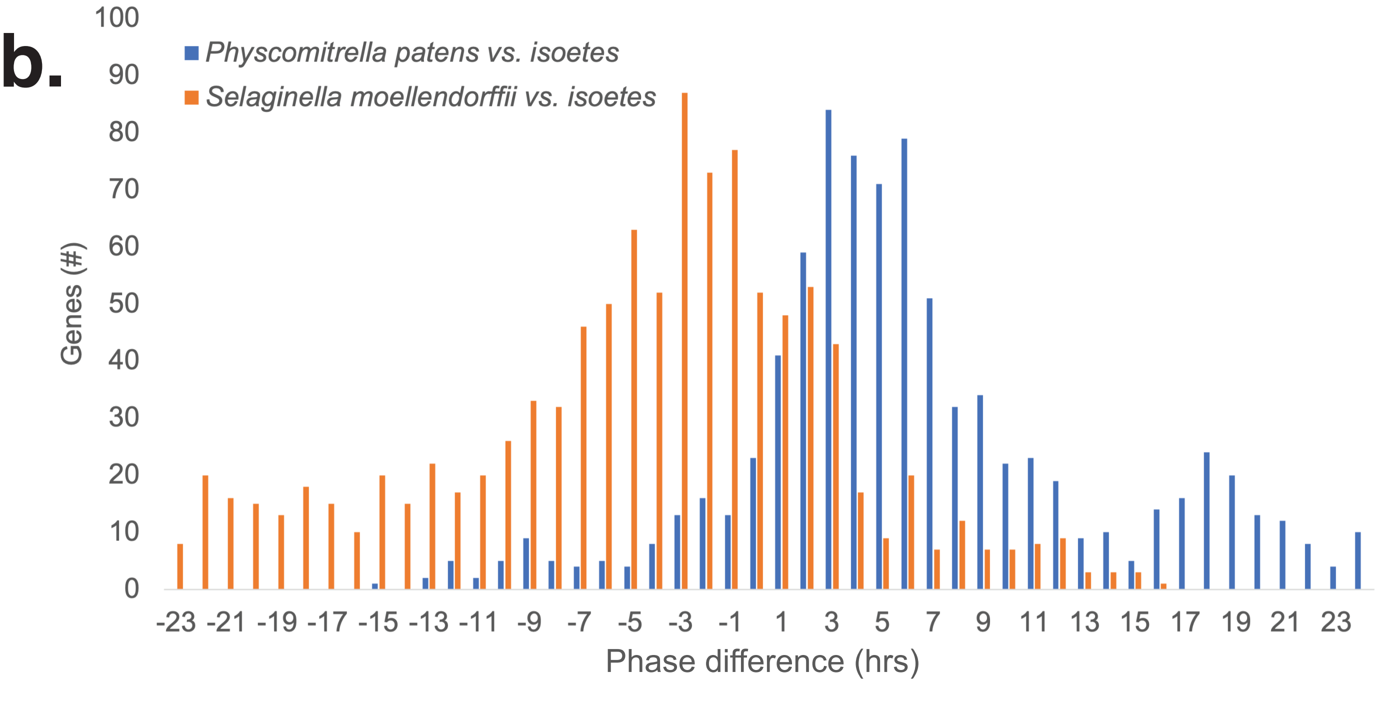

Supplementary Figure 28. Multispecies comparison of global TOD expression. a, Number of genes that cycle with a specific phase of the reciprocal best BLAST hits (RBHs) between *Arabidopsis thaliana* and *Isoetes taiwanensis*. Left column is time (hrs) of peak expression; sum is number of RBHs that cycle; total is the percent of expressed genes that are predicted cycle in that dataset. Larger numbers of cycling genes (green) and fewer (red) are highlighted. LDHC (light/dark; hot/cold); LLHC (continuous light; hot/cold); LDHH (light/dark; continuous hot); long (longdays; 16hrs light/8 hrs dark; continuous hot); short (shortdays; 8hrs light/16 hrs dark; continuous hot); LL_LDHC (continuous light after LDHC); LL_LLHC (continuous light after LLHC); LL12_LDHH (first 2 days in continuous light after LDHH); LL23_LDHH (second and third day in continuous light after LDHH); DD_DDHC (continuous dark after DDHC). **b,** Phase shift between RBHs of *Physcomitrium patens* (blue) and *Selaginella moellendorffii* (orange) with *I. taiwanensis*. Source data are provided as a Source Data file.

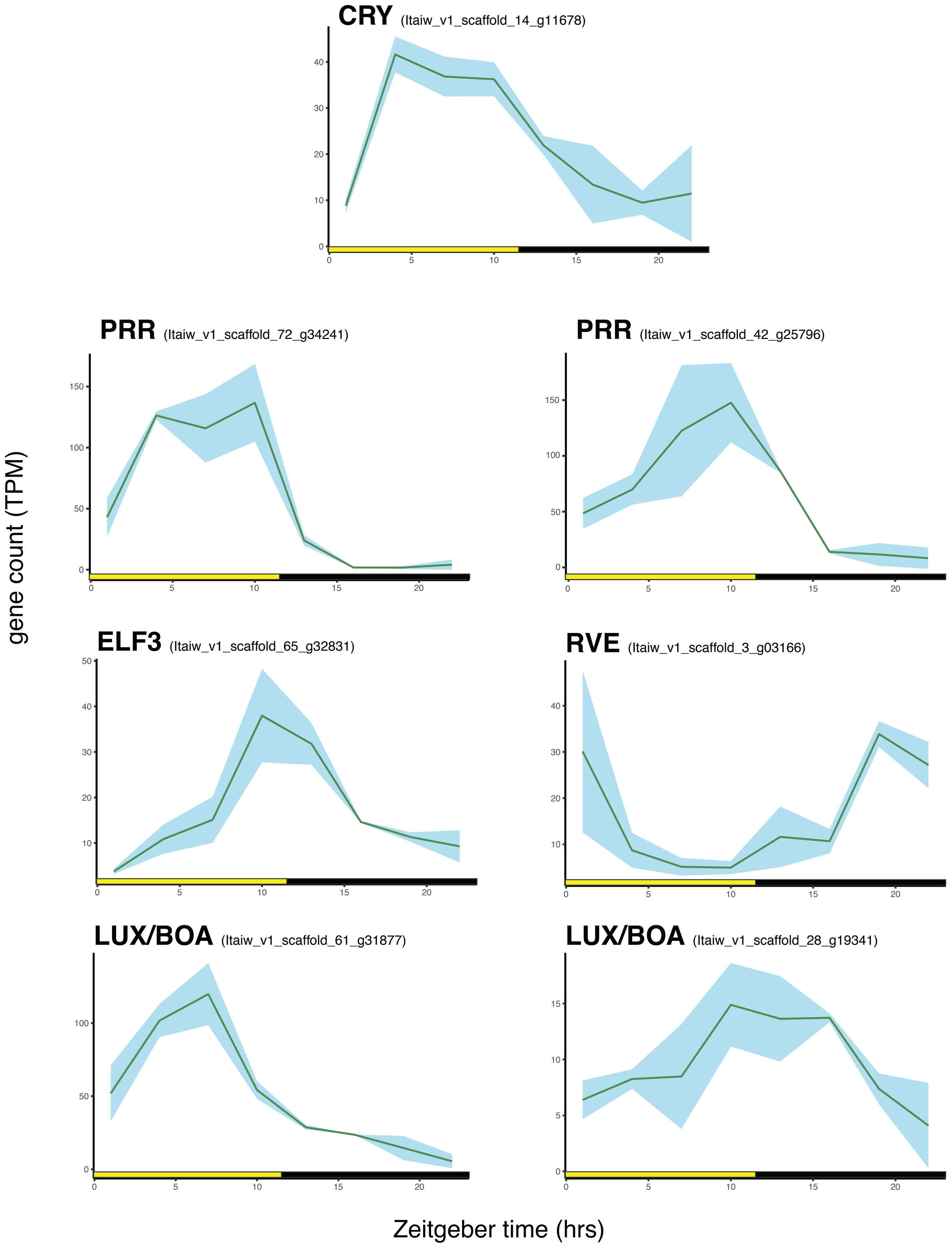

Supplementary Figure S29. Many circadian associated genes exhibit typical cycling behavior in *Isoetes taiwanensis*. Plots showing TOD expression of circadian associated genes in *I. taiwanensis*.). Average of TPM normalized expression data for *I. taiwanensis* is plotted with a shaded ribbon representing the standard deviation. Cycling genes are named for their nearest orthologues in Arabidopsis. Source data are provided in Supplementary Data 2.

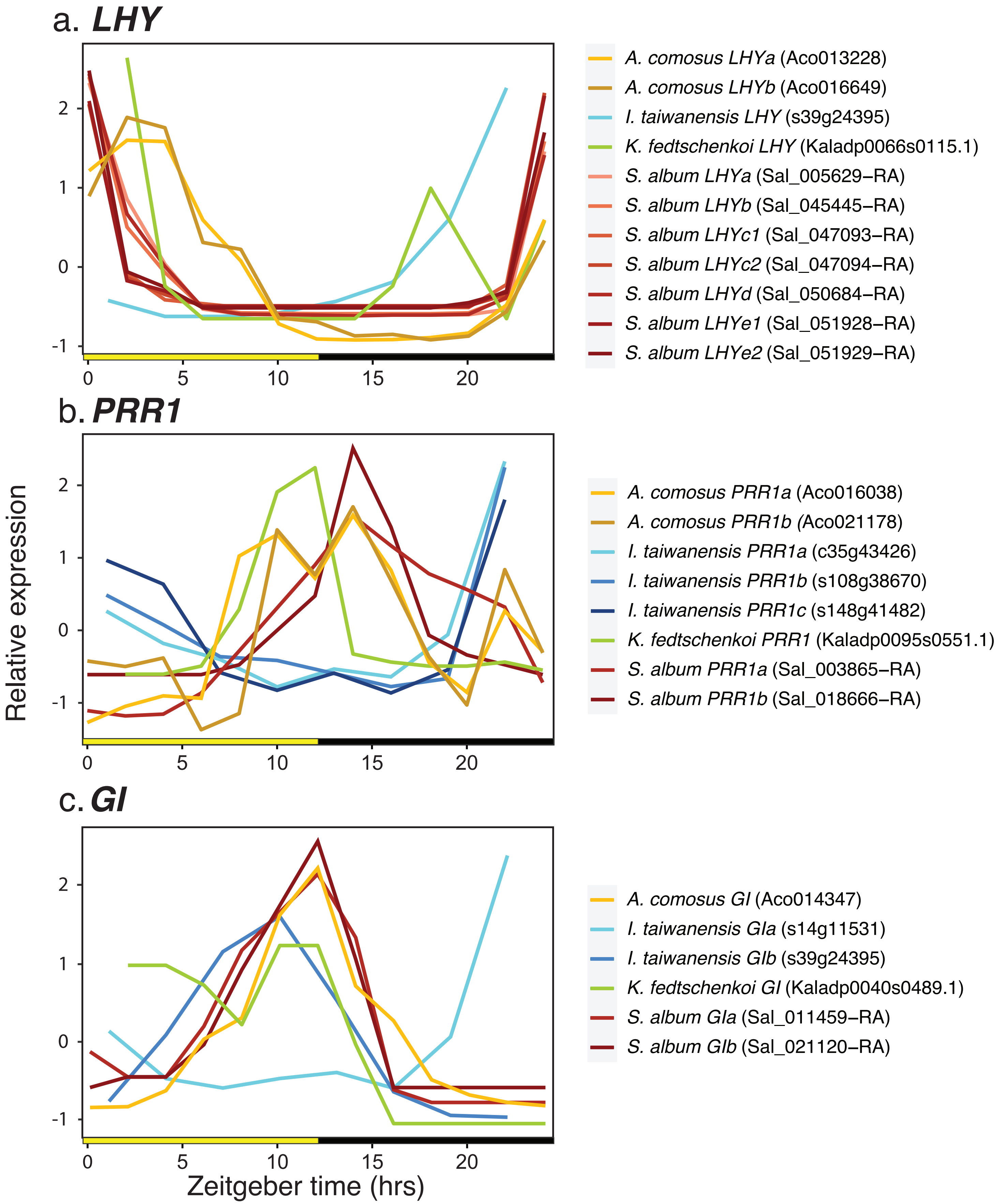

Supplementary Figure 30. Expression of key circadian associated genes is shifted in *I. taiwanensis*. relative to terrestrial CAM plants a, *GIGANTEA* (*GI*), **b,** *PSEUDO-RESPONSE REGULATOR 1* (*PRR1*) **c,** *LATE ELONGATED HYPOCOTYL* (*LHY*) orthologs in *Isoetes* (blue lines), *Ananas* (gold lines), *Sedum* (red lines), and *Kalanchoe* (green line) normalized expression over the day. Day (yellow box); Night (black box); Zeitgeber time (ZT) is the number of hours (hrs) after lights on (0 hrs). Source data are provided for *I. taiwanensis* in Supplementary Data 2 and for other taxa in a Source Data file.

Supplementary References

1. Grewe, F. *et al.* A unique transcriptome: 1782 positions of RNA editing alter 1406 codon identities in mitochondrial mRNAs of the lycophyte Isoetes engelmannii. *Nucleic Acids Res.* **39**, 2890–2902 (2011).
2. Oldenkott, B., Yamaguchi, K., Tsuji-Tsukinoki, S., Knie, N. & Knoop, V. Chloroplast RNA editing going extreme: more than 3400 events of C-to-U editing in the chloroplast transcriptome of the lycophyte Selaginella uncinata. *RNA* **20**, 1499–1506 (2014).
3. Weng, J.-K., Li, X., Stout, J. & Chapple, C. Independent origins of syringyl lignin in vascular plants. *Proc. Natl. Acad. Sci. U. S. A.* **105**, 7887–7892 (2008).
4. Weng, J.-K., Akiyama, T., Ralph, J. & Chapple, C. Independent recruitment of an O-methyltransferase for syringyl lignin biosynthesis in Selaginella moellendorffii. *Plant Cell* **23**, 2708–2724 (2011).
5. Espiñeira, J. M. *et al.* Distribution of lignin monomers and the evolution of lignification among lower plants. *Plant Biol* **13**, 59–68 (2011).
6. Keeley, J. E. CAM photosynthesis in submerged aquatic plants. *Bot. Rev.* **64**, 121–175 (1998).
7. Harris, B. J., Harrison, C. J., Hetherington, A. M. & Williams, T. A. Phylogenomic evidence for the monophyly of bryophytes and the reductive evolution of stomata. *Curr. Biol.* **30**, 2001-2012.e2 (2020).
8. Keeley, J. E. Distribution of diurnal acid metabolism in the genus Isoetes. *Am. J. Bot.* **69**, 254–257 (1982).
9. Rothwell, G. W. & Erwin, D. M. The rhizomorph apex of paurodendron; Implications for homologies among the rooting organs of lycopsida. *Am. J. Bot.* **72**, 86–98 (1985).
10. Hetherington, A. J., Emms, D. M., Kelly, S. & Dolan, L. Gene expression data support the hypothesis that Isoetes rootlets are true roots and not modified leaves. *Sci. Rep.* **10**, 1–10 (2020).
11. Kim, C. M. & Dolan, L. ROOT HAIR DEFECTIVE SIX-LIKE Class I genes promote root hair development in the grass *Brachypodium distachyon*. *PLoS Genet.* **12**, e1006480 (2016).
12. Huang, L., Shi, X., Wang, W., Ryu, K. H. & Schiefelbein, J. Diversification of root hair development genes in vascular plants. *Plant Physiol.* **174**, 1697–1712 (2017).
13. Wai, C. M. *et al.* Time of day and network reprogramming during drought induced CAM photosynthesis in Sedum album. *PLoS Genet.* **15**, e1008209 (2019).
14. Yang, X. *et al.* The Kalanchoë genome provides insights into convergent evolution and building blocks of crassulacean acid metabolism. *Nat. Commun.* **8**, 1899 (2017).
15. Ming, R. *et al.* The pineapple genome and the evolution of CAM photosynthesis. *Nat. Genet.* **47**, 1435–1442 (2015).
16. Mockler, T. C. *et al.* The DIURNAL project: DIURNAL and circadian expression profiling, model-based pattern matching, and promoter analysis. *Cold Spring Harb. Symp. Quant. Biol.* **72**, 353–363 (2007).
17. Michael, T. P. *et al.* Network discovery pipeline elucidates conserved time-of-day–specific cis-regulatory modules. *PLoS Genet.* **4**, e14 (2008).
18. Filichkin, S. A. *et al.* Global profiling of rice and poplar transcriptomes highlights key

conserved circadian-controlled pathways and cis-regulatory modules. *PLoS One* **6**, e16907 (2011).

19. Cronn, R. *et al.* Transcription through the eye of a needle: daily and annual cyclic gene expression variation in Douglas-fir needles. *BMC Genomics* **18**, 558 (2017).
20. MacKinnon, K. J. M., Cole, B. J., Yu, C. & Coomey, J. H. Changes in ambient temperature are the prevailing cue in determining Brachypodium distachyon diurnal gene regulation. *New Phy* 1709–1724 (2020).
21. Ferrari, C. *et al.* Kingdom-wide comparison reveals the evolution of diurnal gene expression in Archaeplastida. *Nat. Commun.* **10**, 737 (2019).
22. Nose, M. & Watanabe, A. Clock genes and diurnal transcriptome dynamics in summer and winter in the gymnosperm Japanese cedar (Cryptomeria japonica (L.f.) D.Don). *BMC Plant Biology* vol. 14 308 (2014).
23. Sato, Y. *et al.* RiceXPro version 3.0: expanding the informatics resource for rice transcriptome. *Nucleic Acids Res.* **41**, D1206-13 (2013).
24. Khan, S., Rowe, S. C. & Harmon, F. G. Coordination of the maize transcriptome by a conserved circadian clock. *BMC Plant Biol.* **10**, 126 (2010).
25. Michael, T. P. *et al.* Genome and time-of-day transcriptome of Wolffia australiana link morphological minimization with gene loss and less growth control. *Genome Res.* **31**, 225–238 (2020).
26. Lai, X. *et al.* Interspecific analysis of diurnal gene regulation in panicoid grasses identifies known and novel regulatory motifs. *BMC Genomics* **21**, 428 (2020).
27. Greenham, K. *et al.* Expansion of the circadian transcriptome in Brassica rapa and genome-wide diversification of paralog expression patterns. *Elife* **9**, (2020).
28. Michael, T. P. *et al.* Enhanced fitness conferred by naturally occurring variation in the circadian clock. *Science* **302**, 1049–1053 (2003).
29. Dodd, A. N. *et al.* Plant circadian clocks increase photosynthesis, growth, survival, and competitive advantage. *Science* **309**, 630–633 (2005).
30. Filichkin, S. A. *et al.* Global profiling of rice and poplar transcriptomes highlights key conserved circadian-controlled pathways and cis-regulatory modules. *PLoS One* **6**, e16907 (2011).
31. McClung, C. R. Circadian clock components offer targets for crop domestication and improvement. *Genes* **12**, (2021).
32. Schaffer, R. *et al.* The late elongated hypocotyl mutation of Arabidopsis disrupts circadian rhythms and the photoperiodic control of flowering. *Cell* **93**, 1219–1229 (1998).
33. Wang, Z. Y. & Tobin, E. M. Constitutive expression of the CIRCADIAN CLOCK ASSOCIATED 1 (CCA1) gene disrupts circadian rhythms and suppresses its own expression. *Cell* **93**, 1207–1217 (1998).
34. McClung, C. R. The Plant Circadian Oscillator. *Biology* **8**, (2019).
35. Strayer, C. *et al.* Cloning of the Arabidopsis clock gene TOC1, an autoregulatory response regulator homolog. *Science* **289**, 768–771 (2000).
36. Matsushika, A., Makino, S., Kojima, M. & Mizuno, T. Circadian waves of expression of the APRR1/TOC1 family of pseudo-response regulators in Arabidopsis thaliana: insight into the plant circadian clock. *Plant Cell Physiol.* **41**, 1002–1012 (2000).
37. Filichkin, S. A. *et al.* Global profiling of rice and poplar transcriptomes highlights key

conserved circadian-controlled pathways and cis-regulatory modules. *PLoS One* **6**, e16907 (2011).

38. Zones, J. M., Blaby, I. K., Merchant, S. S. & Umen, J. G. High-resolution profiling of a synchronized diurnal transcriptome from Chlamydomonas reinhardtii reveals continuous cell and metabolic differentiation. *Plant Cell* **27**, 2743–2769 (2015).
39. Moseley, R. C. *et al.* Conservation and diversification of circadian rhythmicity between a model Crassulacean Acid Metabolism plant Kalanchoë fedtschenkoi and a model C3 photosynthesis plant Arabidopsis thaliana. *Front. Plant Sci.* **9**, (2018).
40. Hu, Y. et al. Crystal structures of a Populus tomentosa 4-coumarate:CoA ligase shed light on its enzymatic mechanisms. *Plant Cell* **22**, 3093–3104 (2010).
41. Pan, H. et al. Structural studies of cinnamoyl-CoA reductase and cinnamyl-alcohol dehydrogenase, key enzymes of monolignol biosynthesis. *Plant Cell* **26**, 3709–3727 (2014).
42. Louie, G. V. et al. Structure-function analyses of a caffeic acid O-methyltransferase from perennial ryegrass reveal the molecular basis for substrate preference. *Plant Cell* **22**, 4114–4127 (2010).
43. Youn, B. et al. Crystal structures and catalytic mechanism of the Arabidopsis cinnamyl alcohol dehydrogenases AtCAD5 and AtCAD4. *Org. Biomol. Chem.* **4**, 1687–1697 (2006).
44. Ferrer, J.-L., Zubieta, C., Dixon, R. A. & Noel, J. P. Crystal structures of alfalfa caffeoyl coenzyme A 3-O-methyltransferase. *Plant Physiol.* **137**, 1009–1017 (2005).
