# Supplementary material for: Underwater CAM photosynthesis elucidated by Isoetes genome
Source: Nat Commun. 2021 Nov 3;12:6348. doi: 10.1038/s41467-021-26644-7 (PMC8566536; doi:10.1038/s41467-021-26644-7)
Supplement: Supplementary file 3 — Description of Additional Supplementary Files [file 41467_2021_26644_MOESM3_ESM.pdf]

Description of Additional Supplementary Files

File Name: Supplementary Data 1
Description: Characterization and quantification of repetitive sequences in the *Isoetes taiwanensis* genome.

File Name: Supplementary Data 2
Description: TPM normalized gene expression values used to generate TOD expression plots for *Isoetes taiwanensis*. Columns are labeled with time of expression followed by replicate number.

File Name: Supplementary Data 3
Description: Locus IDs from multi-species comparisons of TOD specific gene expression.

File Name: Supplementary Data 4
Description: GO term enrichment for genes in *Isoetes taiwanensis* clustered according to TOD expression.

File Name: Supplementary Data 5
Description: Putative novel cis-regulatory elements (CREs) identified in *Isoetes taiwanensis*. While half of the CREs identified by ELEMENT analysis closely matched known transcription factor binding sites in Arabidopsis, many remained unclassified. P-values calculated in TOMTOM.

File Name: Supplementary Data 6
Description: Few known circadian motifs are found upstream of CAM genes with TOD expression in *Isoetes taiwanensis*. P-value and Q-value calculated in FIMO.
